# Empowering communities through One Health and ArtScience: An innovative approach to combat Chagas disease in endemic areas of Minas Gerais, Brazil

**DOI:** 10.1002/puh2.165

**Published:** 2025-02-10

**Authors:** Jonathan Gonçalves‐Oliveira, Catarina Macedo Lopes, Teresa C. M. Gonçalves, Suellen Nunes Sarmento, João Paulo S. O. Correia, Rute H. A. de Sousa, Antônia de Castro Ribeiro, Otília Sarquis, Juliana Almeida‐Silva, Sarah C. dos S. Silva, Ana M. Suarez‐Fontes, Roberto Rodrigues Ferreira, Thallyta M. Vieira, Paulo S. D'Andrea, Luciana R. Garzoni, Marcos A. Vannier‐Santos, Tania C. Araujo‐Jorge

**Affiliations:** ^1^ Laboratory of Hantaviruses and Rickettsiosis Oswaldo Cruz Institute (LHR‐IOC/Fiocruz) Oswaldo Cruz Foundation Rio de Janeiro Rio de Janeiro Brazil; ^2^ Laboratory Interdisciplinary in Entomological Surveillance in Diptera and Hemiptera Oswaldo Cruz Institute (LIVEDIH‐IOC/Fiocruz) Oswaldo Cruz Foundation (Fiocruz) Rio de Janeiro Rio de Janeiro Brazil; ^3^ Laboratory National and International Reference in Triatominae Taxonomy Oswaldo Cruz Institute (LNIRTT‐IOC/FIOCRUZ) Oswaldo Cruz Foundation (Fiocruz) Rio de Janeiro Rio de Janeiro Brazil; ^4^ Laboratory of Biology and Parasitology of Reservoir Wild Mammals Oswaldo Cruz Institute (LABPMR‐IOC/Fiocruz) Oswaldo Cruz Foundation Rio de Janeiro Rio de Janeiro Brazil; ^5^ Laboratory of Ecoepidemiology of Chagas Disease Oswaldo Cruz Institute (LEDOC‐IOC/Fiocruz) Oswaldo Cruz Foundation Rio de Janeiro Rio de Janeiro Brazil; ^6^ Laboratory of Innovations in Therapies Education and Bioproducts Oswaldo Cruz Institute (LITEB‐IOC/Fiocruz) Oswaldo Cruz Foundation (Fiocruz) Rio de Janeiro Rio de Janeiro Brazil; ^7^ Laboratory of Functional Genomics and Bioinformatics Oswaldo Cruz Institute (LAGFB‐IOC/Fiocruz) Oswaldo Cruz Foundation (Fiocruz) Rio de Janeiro Rio de Janeiro Brazil; ^8^ Center for Biological and Health Science Universidade Estadual de Montes Claros Campus Universitário Prof. Darcy Ribeiro Montes Claros Minas Gerais Brazil

**Keywords:** ArtScience, Chagas disease, education, hosts, mammals, One Health, vectors

## Abstract

**Background:**

Chagas disease (CD) is a neglected zoonosis that requires study through the One Health approach, as it involves various aspects of animal, environmental, and human health in its transmission cycle. This disease affects 7 million people in 20 countries in Latin America, resulting in approximately seven thousand deaths annually worldwide. Local knowledge is crucial for developing educational strategies to mitigate the risks of disease transmission, particularly in endemic areas.

**Aims:**

In this study, we present the experience of integrating five stands with complementary subjects on CD, focusing on environmental, biological, and human life factors, as well as its invertebrate and vertebrate hosts.

**Methods:**

This research was conducted as part of the Chagas Express XXI initiative, a social‐educational technology conceived as an imaginary train consisting of six thematic “wagons” (education stations) related to CD. The current study took place in the northern region of the state of Minas Gerais, Brazil, focusing on the activities of wagon 4, which involved 649 participants in two municipalities: Espinosa and Montes Claros, both endemic rural and urban areas for CD.

**Results:**

Participants’ prior knowledge was assessed through a series of questions, and educational mediators engaged in dialog during voluntary sessions. We found that crucial aspects related to the prevention and control of vector‐borne transmissions, such as host and vector diversity, as well as household risks, were neglected in local education activities or unknown to the populations of both municipalities.

**Conclusions:**

This study demonstrates that zoonosis, particularly CD, should be included in basic education and health professional training courses, employing strategies that consider the different socio‐environmental interfaces and aim to improve human, animal, and environmental living conditions.

## Graphical abstract

This study combines One Health and ArtScience to address Chagas disease in Latin America through Chagas Express XXI in Minas Gerais, Brazil. Six workshops revealed significant gaps in local understanding of transmission and control, emphasizing the need for education tailored to endemic socio‐environmental contexts.

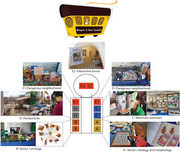

## INTRODUCTION

Chagas disease (CD), a zoonosis caused by the parasite *Trypanosoma cruzi*, combines biomedical, epidemiological, sociocultural, and political factors in maintaining its zoonotic cycle [1]. CD affects around 6–8 million people in 21 countries in Latin America, with approximately 12,000 deaths annually across the world [2]. In Brazil, the number of chronically affected persons is estimated for 2020 as 1365,585–3213,142 [3], the highest number in the world, and this disease represents a threat to public health. In recent years, the persistence of cases associated with vector transmission has been notable, as well as the increase, mainly, of those correlated with oral transmission [4]. These conditions demand educational strategies in the light of the One Health approach, which allows the understanding of health problems in a holistic and multisectoral way, discussing how these factors influence the dynamics of CD in certain environments [5]. Sanmartino et al. [1] warn of the need for more studies that address vector recognition, the participation of reservoirs mammal's, the importance of the environment in the transmission of *T. cruzi*, and how formal and nonformal spaces for education should facilitate the understanding of the CD scenario, highlighting the sociocultural aspects of the studied places.

CD is a vector‐borne disease: For the infection of hosts to occur, it is necessary for the direct or indirect participation of vectors in the maintenance of the parasite. Vector control then becomes one of the essential strategies to contain CD transmission [6]. Actions to control CD vector insects in endemic areas involve chiefly the use of insecticide chemicals without considering other health conditions, such as poor hygiene and home environment disorder, increasing risk factors by providing innumerous insect shelters, thus allowing, or favoring colonization [7].

Another aspect to be considered in endemic areas is the awareness of segments of society concerning the knowledge of prevention measures carried out continuously, in partnership with local managers in the areas of health and education. It is relevant to avoid the colonization of these insects’ domestic environment and to maintain full entomological surveillance. In Argentina and Brazil, studies were carried out to raise awareness of the population about the disease, its symptoms, forms of transmission, and vector insects. It is then recommended that the population should actively contribute to prevention and control, and in the case of the Brazilian territory, to strengthen the Unified Health System (Sistema Único de Saúde) in response to these demands [8, 9, 10].

To approach these issues, we developed a new ArtScience social technology for health and science education named “Chagas Express XXI” (CE) that was conceived for CD literacy and surveillance interventions [11]. CE is an “imaginary train” divided into six thematic stations the so‐called wagons: (i) Associations—for conversations with the affected people, where it is possible to exchange experiences and foster civilian organization; (ii) Discovers—covering the history of the discovery of CD, diagnosis and treatment; (iii) Playful Universe, with games and activities to learn about the social and biological determination of the disease; (iv) Home and One Health, addressing hosts and environments; (v) Well‐being, perception of health in mental, emotional, social, and physical aspects; and (vi) Listening to the population about the experience at CE. In wagon 4, we organized five thematic workshops approaching One Health, articulating entomological, environmental, and animal and human health information concerning the complexity of CD transmission cycle [11, 12].

The aim of the present study was to describe the creative conception of wagon 4, all its elements, and the perception of the participants during dialogs with the educational mediators that provoked integration discussions concerning animal, environment, and human health and estimating local knowledge about vectors, reservoirs, risk scenarios and peridomicile and domicile dangers. We expected that combining One Health [13] with ArtScience [11, 12] and approaches to disseminate science information about CD and to dialogically interact with the population living in endemic areas, we will help to foster knowledge construction for a better understanding of the complex environmental, biological, and human life factors, as well as its invertebrate and vertebrate hosts implicated in transmission and reemergence.

## METHODS

### Conception and study areas

CE is a social technology that was cocreated and co‐mediated by students, scientists, and, worthily, by people affected by CD, proposing more than 40 educational activities disposed in stands, games, laboratory events, and exhibitions [11]. CE is also an itinerant exhibition and was performed, validated, and evaluated in July 2019 in Brazilian schools and universities at the north of state of Minas Gerais, a CD endemic area, fully described elsewhere, including the table with the ArtScience activities [11].

The CE “imaginary train” alludes to CD discovery. Carlos Chagas worked in a railway wagon while discovering the parasite's full life cycle. From the six thematic modules, the “train wagons,” the so‐called “wagon 4: Home and One Health” was devoted to the recognition of CD vectors and mammal reservoirs, raising awareness about the natural risks of CD transmission. During the CE field validation test, wagon 4 presented educational activities in four endemic cities and the state capital [11]. We chose two of them to invite the participants to answer questions to inform their previous knowledge regarding One Health approach for CD. The other six CE wagons approached different subjects, including social determinants of health and social organization of affected people, among others [11]. In the entry station, all the participants were registered with main personal data, including sex, age, educational level, and living region. They received their personal badge and answered to four brief questions concerning knowledge about CD, as reported for the five different municipalities [11].

Espinosa and Montes Claros were the two cities where the present study was conducted, attracting, respectively, 1145 and 352 participants [11]. During the wagon 4 proposed activities, 411 persons voluntarily answered the questionaries in Espinosa (35%) and 231 in Montes Claros (65%). These two municipalities were chosen during the preliminary study setting meetings with regional health managers and stakeholders who indicated hot spots of chronic cases and entomological surveillance indicators [14]. Montes Claros is the largest city (>400,000 habitants) in the northern region of Minas Gerais state, located 422 km from the state capital Belo Horizonte. CE activities were presented in the State University hall, and community health agents were previously motivated to come in minibuses with people living in their respective areas of surveillance. Espinosa is a ∼30,000 habitants city located 277 km north from Montes Claros, almost in the Minas Gerais division with Bahia state, an important endemic region for CD. CE was presented in an elementary school in an accessible neighborhood chosen by the Municipality Health Secretary. In this city, people were motivated both by their community health agents and by local radio calls. In both cities, the exhibition lasted 2 days. Details of the geographical, demographic and epidemiological characteristics of these two municipalities are described in our first study (see Table 2 in Araujo‐Jorge et al. [11]), where differences in the age and education level were also noted between them.

### Theoretical framework in ArtScience and One Health

Mediators in the exhibition stands used the ArtScience approach to create the materials presented, to promote creativity, and to strengthen nonformal education‐based learning. This theoretical framework has been described in Brazil in the last 30 years [12, 15] and is grounded on the thesis of the ArtScience Manifesto [16], using the 13 cognitive categories described by Robert and Michèle Root–Bernstein [17] as inductors of imagination and creativity and as an analytical matrix. The “wagon 4” stands were conceived involving aspects of the health of humans and of the domestic/wild environments, using photographs, models, draws, taxidermized mammals, preserved insects, banners, and software. The diagnosis of zoonotic infections and the corresponding health interventions in the territory integrated all these subjects in the concept of One Health [18, 19]. This concept refers to a set of initiatives that aims to integrate professionals from different areas of health and research to cooperate in the development of strategies for coping and mitigating emerging and reemerging diseases [5, 20].

### Description of Chagas Express XXI wagon 4 structure, content, and dynamics—“Home and One Health”

The exhibitions prepared to CE wagon 4 aimed to show how the CD parasite is present in the natural environment and its pathways to reach humans unveiling commonly unknown risks. The observed phenomena were as follows: (i) *environmental drivers*, such as the relationship between conservation of the natural environment and infection in mammalian hosts, and the gradient of anthropization that facilitates the occurrence of potential CD vectors at the interface with the domestic environment; (ii) *biological drivers*, such as the biology of insect's vectors involved in the CD dynamics cycle and the species of vectors typical of the northern region of Minas Gerais and known by the community, as well as the biology of wild mammals involved in the maintenance of the natural zoonotic cycle; and (iii) *human life drivers*, such as the situation of houses close to the forest area, conditions and risk situations in the peridomicile that promote the presence and permanence of vectors in the houses and, consequently, the transmission of the parasite to the residents and domiciled animals, becoming a risky environment in the framework of CD control and transmission. The oral transmission was not included in this exhibition, because the main route of transmission in these areas is related to vectorial transmission.

**FIGURE 1:**
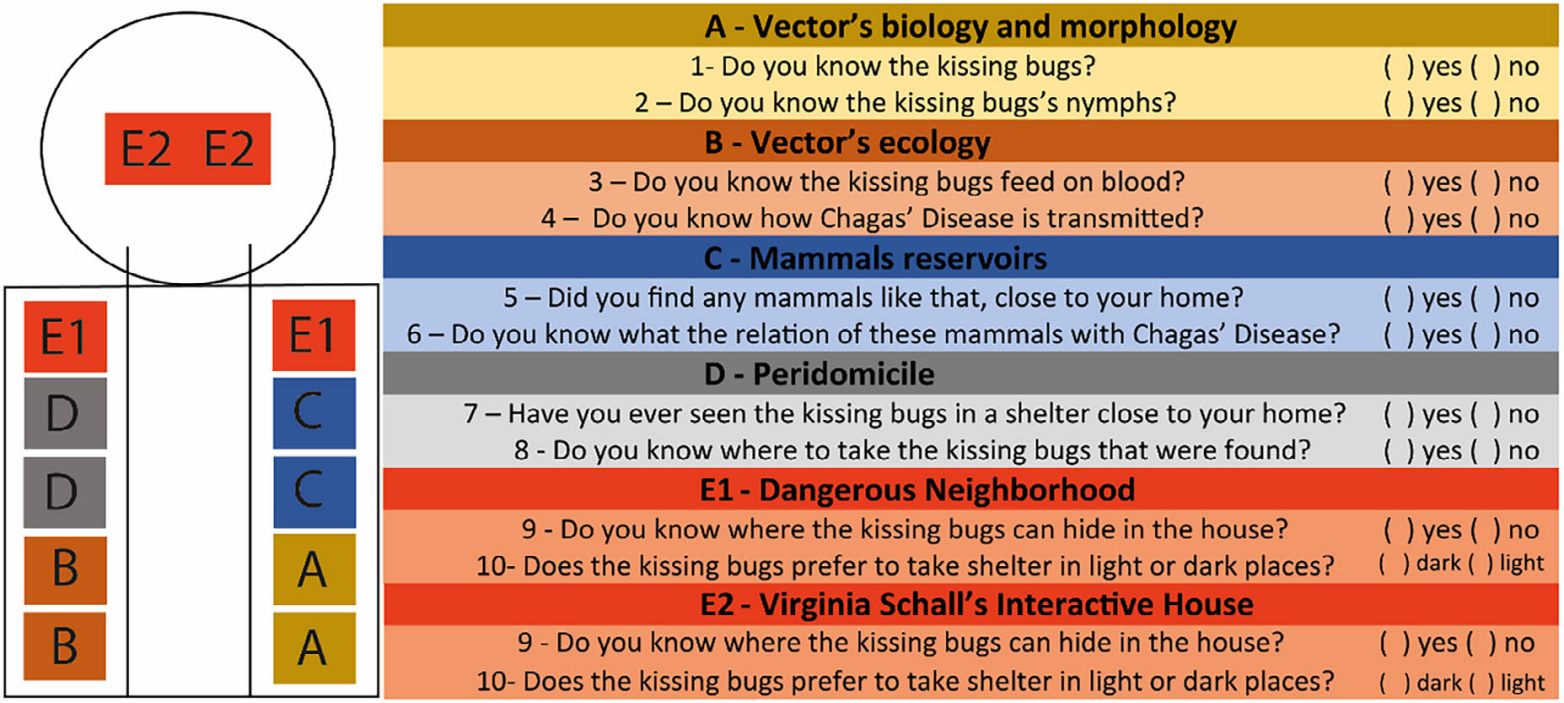
Layout of both sides of the exhibition stand in the wagon 4 of Chagas Express XXI (on the left) and questions proposed in each stage. The letters in the layout correspond to presenting tables with the materials disposable for observation and/or touching; the concise questions (on the right) that the mediators asked the participants were previously standardized and accorded with the team and were proposed before establishing a dialog to actively explore the education materials and to foster finding the answers to the provocative questions.

In this context, we organized a double exhibition stand, with left and right tables (see images in Supplementary Data S1), proposing an integrative model for the presentations. The visitants enter in a linear circuit from A to E and interact with the ArtScience materials organized in two supporting tables for each stand in Figure 1 with redundant proposals for each subject (two different activities for A, for B, and so on). All activities of stands A–E are described and shown in images and banners in Supplementary Data S1.

Concerning the dynamics of the approach, in each wagon 4 stand (Figure 1A–E), one mediator invited the participants to observe, discover, and discuss the proposed subjects and asked them orally the proposed questions (Figure 1A–E, right panel). The questionary aimed both to detect the original knowledge of the participants and to establish a dialog with the mediators to foster discoveries concerning the subjects as well as to provoke the construction of new knowledge anchored in the participant's previous perceptions. The participants choose freely to participate in the activities of wagon 4 and spent about 15–40 min in the exhibition. The answers were considered if the participant completed the activity voluntarily, were registered in the field notebook of each mediator, and were consolidated later by the team coordinator (author 1: JG‐O). The previous team training was important to converge the objectives of the questionary and the consequent results. Each participant interacted with as many stand workshops as they were interested to, and the numbers of answers were expected to be different for each stand. Family visits helped to get oral answers even from small children.

### Quantitative and qualitative analysis

In the quantitative analysis, the number of answers obtained in wagon 4 was compiled, and their frequency was calculated in the municipalities where the questions were proposed. In the qualitative analysis, the evaluation of the answers was performed at each stand, considering that each stand had two qualitative questions asked in the linear visiting circuit. Tables with the 10 questions and the total number of answers were segmented by categories (Yes/No), by municipality (Espinosa/Montes Claros), by age range (children/adults), and, for children and teenagers, by Brazilian education system levels (preschool, elementary, and high school). “Don´t Know” answers were considered “No.” The questions were characterized as “Transmission” or “Control” based on the content of possible responses. The questions are presented in Figure 1, and in the tables and chi‐square analysis of answers, distribution was performed to compare differences between the two municipalities. For qualitative analysis, we transcribed and translated all the statements that some participants spontaneously recorded, which are available in the project YouTube channel (https://www.youtube.com/@ExpressoChagas), selected those that evidenced changes in health professional perceptions and knowledge, and prepared a word cloud to stress the most frequent terms used by the participants, to go beyond, and to complement the quantitative “yes or no” approach.

## RESULTS

### Complete description of wagon 4 activities

**FIGURE 2:**
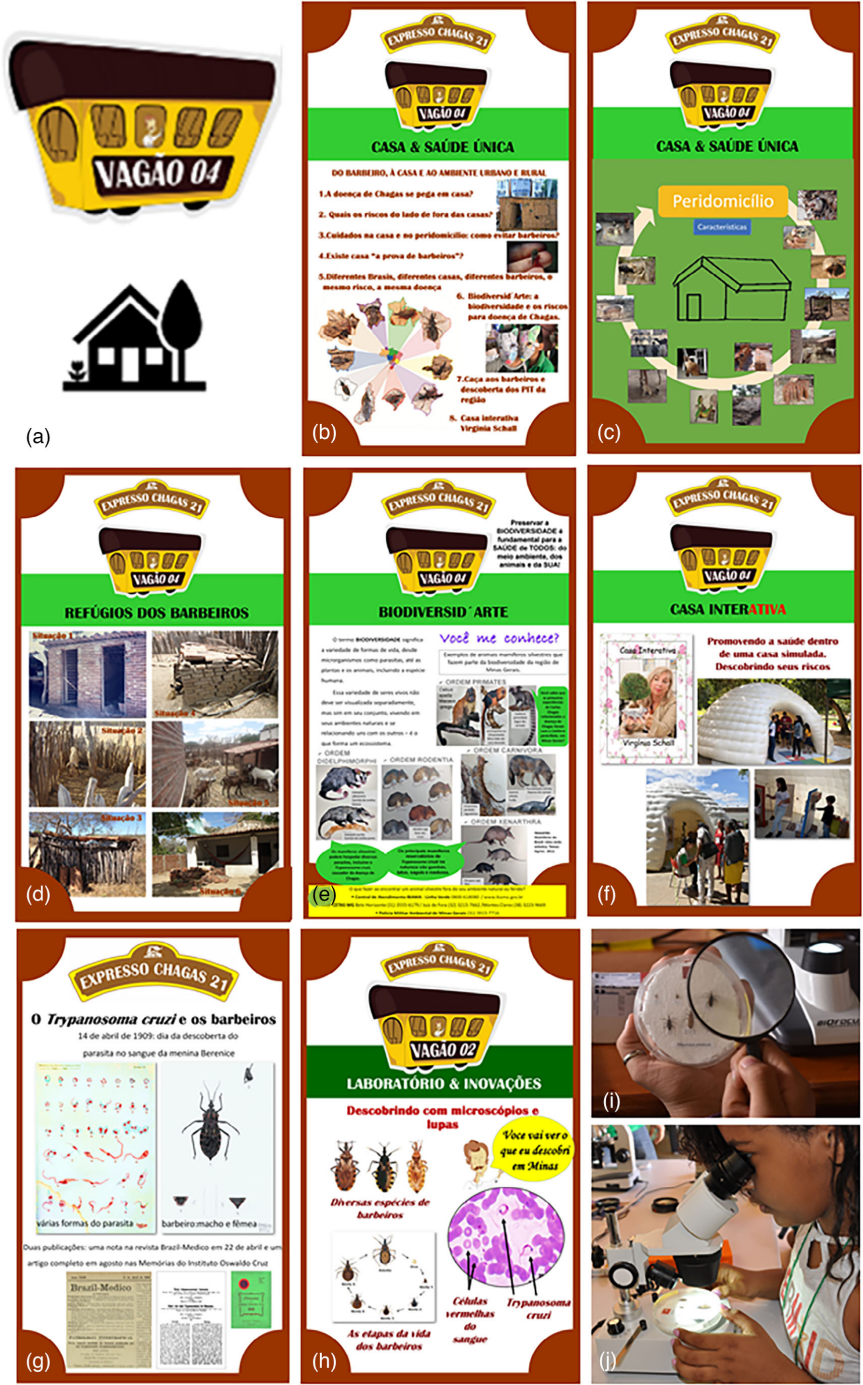
Standard banners prepared for wagon 4 exhibition of Chagas Express XXI initiative (A–F) and other previous stations (G and H). Images are originals in Portuguese, freely available in pdf at: https://www.arca.fiocruz.br/handle/icict/41554. In (a), the wagon 4 logo and symbol (see the visual design in https://journals.plos.org/plosntds/article?id https://doi.org/10.1371/journal.pntd.0009534). (b) Introducing banner entitled “From kissing bugs to the house, urban and country environments,” presenting provoking questions: 1—Do we get Chagas disease at home? 2—What are the risks outside the house? 3—Taking care of the house and peridomicile: how to avoid the kissing bugs? 4—Is there a vector‐proof house? 5—Different Brazil regions, different houses, different kissing bugs: the same risks, the same diseases? 6—Biodiversity'Art: the biodiversity and risks for Chagas disease, 7—Kissing bugs hunting and the discovery of regional PIT (Posto de Informação de Triatomíneos—Triatomine Information Posts), 8—Virginia Schall interactive house. (c) Peridomicile profiles; (d) V = Kissing bugs hiding places; (e) Biodiversity'Art: Do you know me? (f) Interactive House: promoting health inside a simulated house. Discovering the risks. Parts (g) and (h) show triatomines presented in the exhibition in previous stages: (g) the banner presenting the original paper from Carlos Chagas, published in 1909. (h) The banner of wagon 2, entitled “Discoveries and laboratory,” presenting the three triatomine species that are vectors for *Trypanosoma cruzi*, and a micrograph of the parasite seen in stained blood smears as Chagas saw them in 1907–1909 in his train laboratory in the city of Lassance. Participants observing kissing bugs under a hand magnifier (i) and a laboratory table lens (j). Only in wagon 2 these instruments were available to manipulation by the visiting public of Chagas Express XXI exhibition.

**FIGURE 3:**
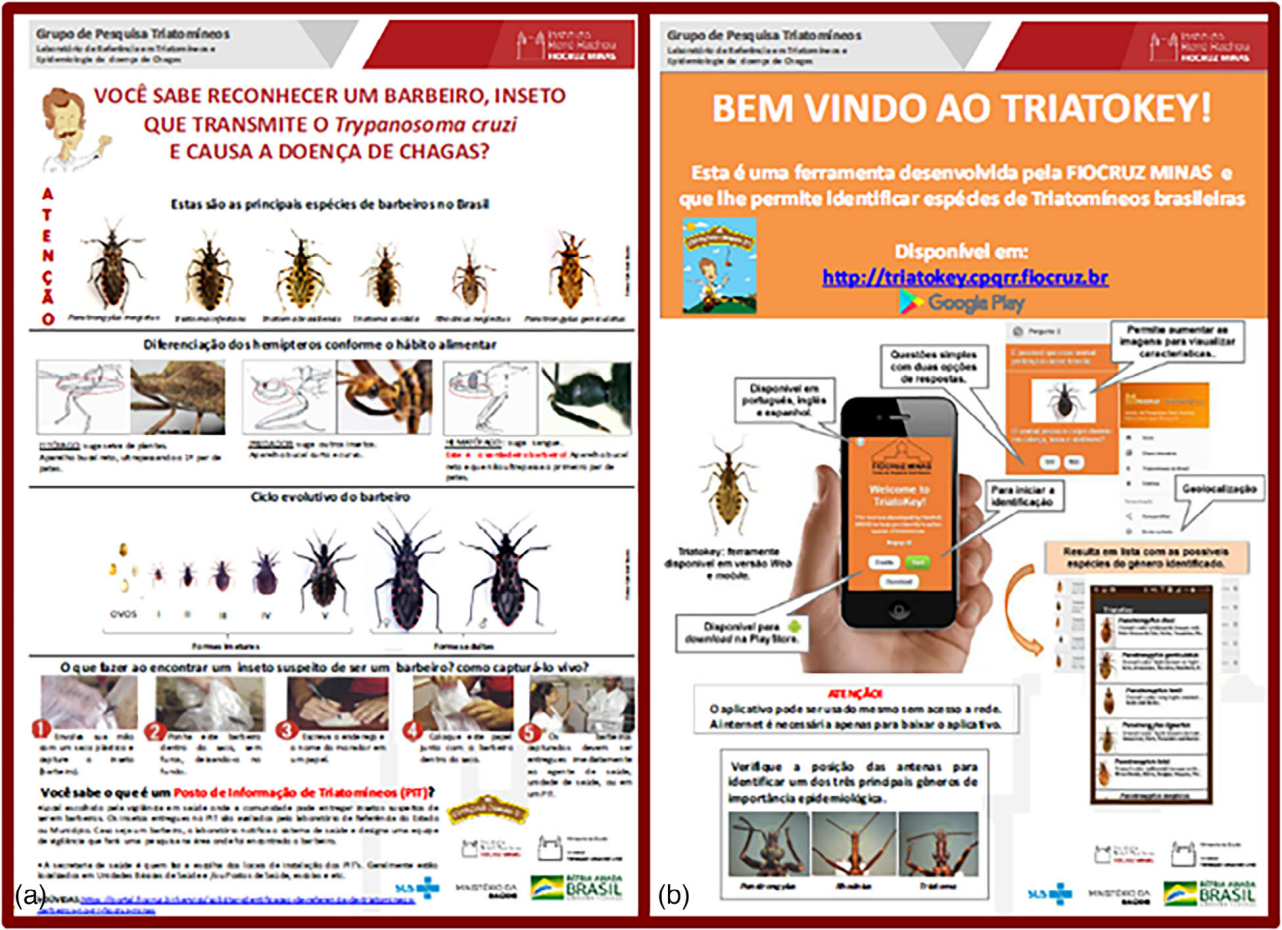
Two banners prepared for wagon 4 exhibition of Chagas Express XXI by the science group that developed the Triatokey app (available at http://chagas.fiocruz.br/). (A) Diversity and morphological characteristics of the regional Chagas disease vectors, including instructions to local procedures to inform health authorities about the presence of a triatomine found in the house or its peridomicile. (B) Tutorial to access the Triatokey app, available at http://triatokey.cpqrr.fiocruz.br.

The art setting of wagon 4 (Figure 2A) and its main five banners (Figure 2B–F) are shown in Figure 2. They complemented the general description of wagon 4 presented in our first study (see Table 1 and Figure 3D in Araujo‐Jorge et al. [11]). These subjects linked wagon 4 activities with those presented in wagon 2, where the major discovery of Carlos Chagas was exposed in the banner shown in Figure 2G, followed by practical activities allowing the observation of preserved kissing bugs and fixed bloodstream parasites, both in banner images (Figure 2H) and under magnifier lenses (Figure 2I,J). Two other banners stimulated the participants to inform the finding of a kissing bug and to try to identify it (Figure 3A,B). They were designed to disseminate the *Triatomine Information Posts* that are a way to promote popular entomological surveillance as a public health policy in Brazil [21], and the *Triatokey* web and mobile tool for the identification of Brazilian triatomine species [22]. The experiences of each stand are described in Supplementary Data S2.

**TABLE 1:** All respondents from Espinosa and Montes Claros municipality, Minas Gerais state.

		Espinosa (total participants = 411)					Montes Claros (total participants = 238)				
Category	All the participants	Age (mean)	Respondents *n* (%) 100% = 411	Answers Yes (%)	Answers No (%)	No answer (N/A)	Age (mean)	Respondents *n* (%) 100% = 238	Answers Yes (%)	Answers No (%)	No answer (N/A)
Transmission	A: Q1—Do you know the kissing bugs?	38.7	138 (34)	118 (86)	20 (14)	273	36.9	74 (31)	50 (68)	24 (32)	164
Transmission	A: Q2—Do you now the kissing bugs’ nymphs?			71 (52)	67 (48)				23 (31)	51 (69)	
Control	B: Q3—Do you know the kissing bugs feed on blood?	38.6	228 (55)	173 (75)	55 (25)	183	36.6	118 (50)	96 (81)	22 (19)	120
Transmission	B: Q4—Do you know how Chagas disease is transmitted?			67 (30)	161 (70)				65 (55)	53 (45)	
Control	C: Q5—Did you find any mammals like that, close to your home?	38.8	170 (41)	145 (85)	25 (15)	241	36.8	154 (65)	96 (62)	58 (38)	84
Transmission	C: Q6—Do you know what the relation of these mammals with Chagas disease?			41 (24)	129 (76)				39 (25)	115 (75)	
Transmission	D: Q7—Have you ever seen the kissing bugs in a shelter close to your home?	38.9	105 (26)	61 (58)	44 (42)	306	36.9	59 (25)	19 (32)	40 (68)	179
Control	D: Q8—Do you know where to take the kissing bugs that were found?			68 (64)	37 (36)				37 (68)	22 (32)	
Transmission	E1: Q9—Do you know where the kissing bugs can hide in the house?	38.5	37 (9)	31 (83)	6 (17)	374	37.0	23 (10)	21 (91)	2 (9)	215
Control	E1: Q10—Does the kissing bugs prefer to take shelter in light or dark places?			27 (73)	10 (27)				19 (82)	4 (18)	
Transmission	E2: Q9—Do you know where the kissing bugs can hide in the house?	38.6	67 (16)	38 (57)	29 (43)	344	37.0	22 (9)	16 (73)	6 (27)	216
Control	E2: Q10—Does the kissing bugs prefer to take shelter in light or dark places?			41 (61)	26 (39)				20 (90)	2 (10)	
	Total number of answers (100%)		1490 (100)	881 (59)	609 (41)			900 (100)	501 (56)	399 (44)	1956
Transmission	Transmission related knowledge (7 questions, 100%)		883 (100)	427 (48)	456 (52)				233 (44)	291 (56)	
Control	Control related knowledge (5 questions, 100%)		607 (100)	454 (75)	153 (25)				268 (71)	108 (29)	

### Age profile of participants

**FIGURE 4:**
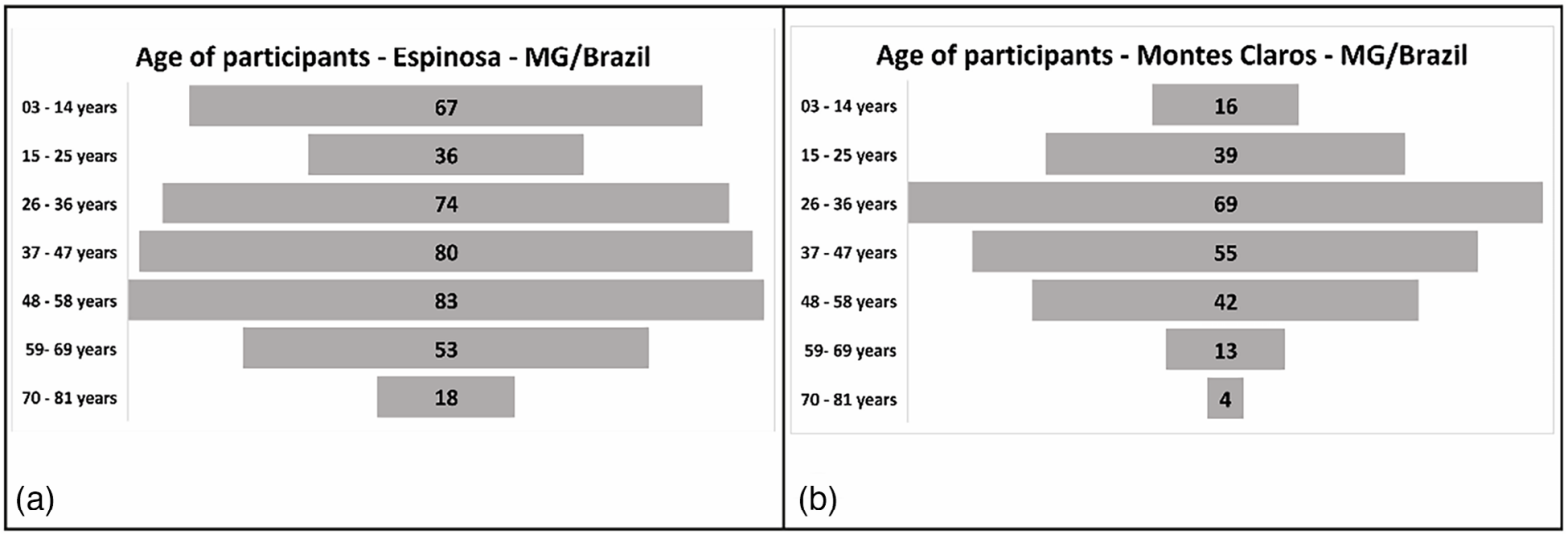
Age distribution of participants in wagon 4: (a) municipality of Espinosa; (b) municipality of Montes Claros. *Note*: Significant differences between the two cities were found in age ranges 3–14; 15–25; 26–36; and 58–68.

The age ranges of the participants in two municipalities were different, ranging from 3 to 81 years old (Figure 4). Children and teenagers from 3 to 14 years old were significantly more frequent in Espinosa (16%) than in Montes Claros (7%). On the contrary, adults from 26 to 36 years old were significantly more frequent in Montes Claros (29%, 69 out of 238) than in Espinosa (18%, 74 out of 411). The frequency of older visitants (58–81 years old) was significantly higher in Espinosa (17%) than in Montes Claros (7%). This confirmed data obtained in the study with all the 1145 participants of Espinosa and 352 in Montes Claros [11], showing that families with children and aged people attended strongly to the activities in Espinosa and adults, especially those with a higher education level, attended CE workshops in Montes Claros. This profile is important for the subsequent analysis of the answers obtained in the five stands of wagon 4.

### Knowledge of wagon 4 participants during educational activities: study with adults

After entering the CE exhibition at the “Lassance Station” [see Araujo‐Jorge et al. [11] for details], the only mandatory exhibition for all participants was wagon 1, presented by CD‐affected individuals who are engaged in CD associations. Subsequently, the participants’ interest and motivation to explore the contents of the other five wagons, either by visiting them as a fair stand or by interacting with the workshop mediators in a dialogical way, revealed the main attraction factor for the people who attended the CE exhibition. Table 1 shows the quantitative results compiled in the two municipalities. In Espinosa, 411 individuals participated in the “Home and One Health” wagon 4 stands and answered the proposed questions. This number represented 35% (411/1145) of the total number of participants in the CE initiative in the city. In Montes Claros, 238 individuals participated in the stand, and this number represented 67% (238/352) of the total number of participants in the initiative in that city.

Wagon 4 had a higher demand in Montes Claros (67%) than in Espinosa (35%), and in both cities, the average age range of respondents was similar, unlike the distribution of the general age range (Figure 4), which shows great interest in wagon 4 among children in Espinosa. Nevertheless, the strategy of questioning participants segmented by stand was successful in collecting over 2000 responses in total for the study (1490 in Espinosa and 900 in Montes Claros). The percentage of total positive and negative responses was similar in both cities and highlighted knowledge gaps. The study captured more responses at stands A–D, and only at stand E, the frequency was lower. The large number of nonrespondents (NA column) was expected due to the nature of the exhibition as a science fair and the linearity of the circuit. Out of the 12 questions, 7 addressed concepts related to transmission and 5 to control, which is why we did a summation of the responses for cumulative analysis. About 50% of the participants from both cities have knowledge gaps related to transmission and 25% in relation to control.

In Espinosa and Montes Claros, although most respondents are aware of the kissing bugs and their blood‐feeding habits, only a minority is familiar with the nymphs (stands A and B). In Espinosa, two thirds of the respondents did not associate this knowledge with the transmission of CD (161/228), whereas in Montes Claros, the majority did make this association. Stand C, in both municipalities, shows that participants were aware of or have seen the mammalian reservoirs but were unaware of their connection with CD. This indicates a lack of knowledge about the involvement of wild mammals in the CD cycle. Regarding the risky environments due to hiding places of kissing bugs (stand D), it was observed that in Espinosa, a more rural area, these vectors were found in hiding spots near the participants’ homes, unlike in Montes Claros, which is a more urban area. In both areas, participants reported knowing where to take the kissing bugs if found near their homes. However, the large number of positive responses regarding sighting these insects near their homes signals the circulation of these vectors close to residential areas, regardless of being rural or urban. At stands E1 and E2, in both municipalities, participants reported knowing where the kissing bugs can hide inside homes and that these bugs prefer darker environments. Based on the classification by transmission and control categories, in both municipalities, and grounded on the cumulative of positive/correct responses, it could be observed that questions related to transmission were less representative than those related to the control of CD.

### Knowledge of children and teenagers participating in wagon 4 during educational activities

The children from Espinosa who responded to the questionnaires represented 19% of the total sample, with the larger number of respondents aged between 6 and 14 years (see Supplementary Data S3. Table 1). In Montes Claros, the quantity was even smaller (Supplementary Data S3. Table 2), representing 3% of the total sample, with children in the same age range. In Espinosa, respondent children under the age of 6 visited stands B and E, and those over 14 did not visit stand D. In Montes Claros, only 1 child under 6 and 1 child over 14 answered the questions, and only at stand C.

Based on the group of children aged 6–14 in Espinosa, the majority apparently had a limited knowledge about CD and its general aspects. Most did not know about the kissing bugs, the nymphs, and their relationship with the transmission of CD, which contrasts with the fact that they knew these bugs feed on blood. Regarding mammalian hosts, they had seen them near their residences but were also unaware of their role in the parasite cycle. Among the respondents, it was also noticeable that they did not know about the hiding places of the kissing bugs and where to take them if found. At stands E1 and E2, the interaction with the models and houses likely contributed to the positive responses regarding finding kissing bugs in the residences. Apparently, in this group, information about Chagas transmission is more limited than that related to control. The largest responding group in Montes Claros was also children aged 6–14, even though the frequency was lower. In this group, it was noted that information about the kissing bugs, nymphs, their hiding places, and their feeding habits was limited. Most were aware of the mammalian hosts but also did not know about their role in the transmission of CD.

### Qualitative statements of participants after wagon 4 educational activities

Wagon 6 was the end point where the participants spontaneously led their statements about the perceptions, feelings, and discoveries they got in the whole exhibition. Some were noted, and others were video recorded. We selected four to present perceptions related to wagon 4, stated by control agents of endemics. These and other original records can be observed at https://www.youtube.com/@ExpressoChagas), in Portuguese.

1. “We wanted to thank you for everything, for your attention, for your affection, for the information you gave us. We arrived here with our eyes closed and we are leaving with great light so that we can reach the rural areas, wherever we arrive, with the message, with the knowledge, so that we can pass it on to people correctly. When we arrived here as Agents, we had no idea if I put my hand on the wall in the barber's feces, I could be contracting the disease. Today I will arrive at the resident's house so I can guide him 100% correctly.”
2. “Now I know how to differentiate a kissing bug from another predator, for bedbugs, for beetles, there is a huge difference. So, I learned a lot about this, I know how to differentiate, I know where it is, where their natural habitat is and where they are migrating to. I acquired a lot of knowledge there, and as I work in the health sector, I want to pass on this knowledge to the people I deal with on a daily basis.”
3. “It was an additional knowledge acquired, because often we do the combat part, but the treatment part, how it is done, what steps we didn't have this knowledge, we had the basic knowledge, so I improved. This makes me an increasingly capable professional and I would like to thank the team, the entire team that came to bring this knowledge to us, knowledge is wealth.”
4. “Today here in the wagon, I received information that never existed at the Zoonosis Control Center. As an agent of endemics control, I had no information and training on Chagas disease as great as I got here. Today I learned all the phases of barbering, transmission. Places where everything about barbering can be found were reviewed here today. And this helps us in practice and in our day‐to‐day work. We hope that the authorities will come and train us with other diseases that the agents must work with.”

**FIGURE 5:**
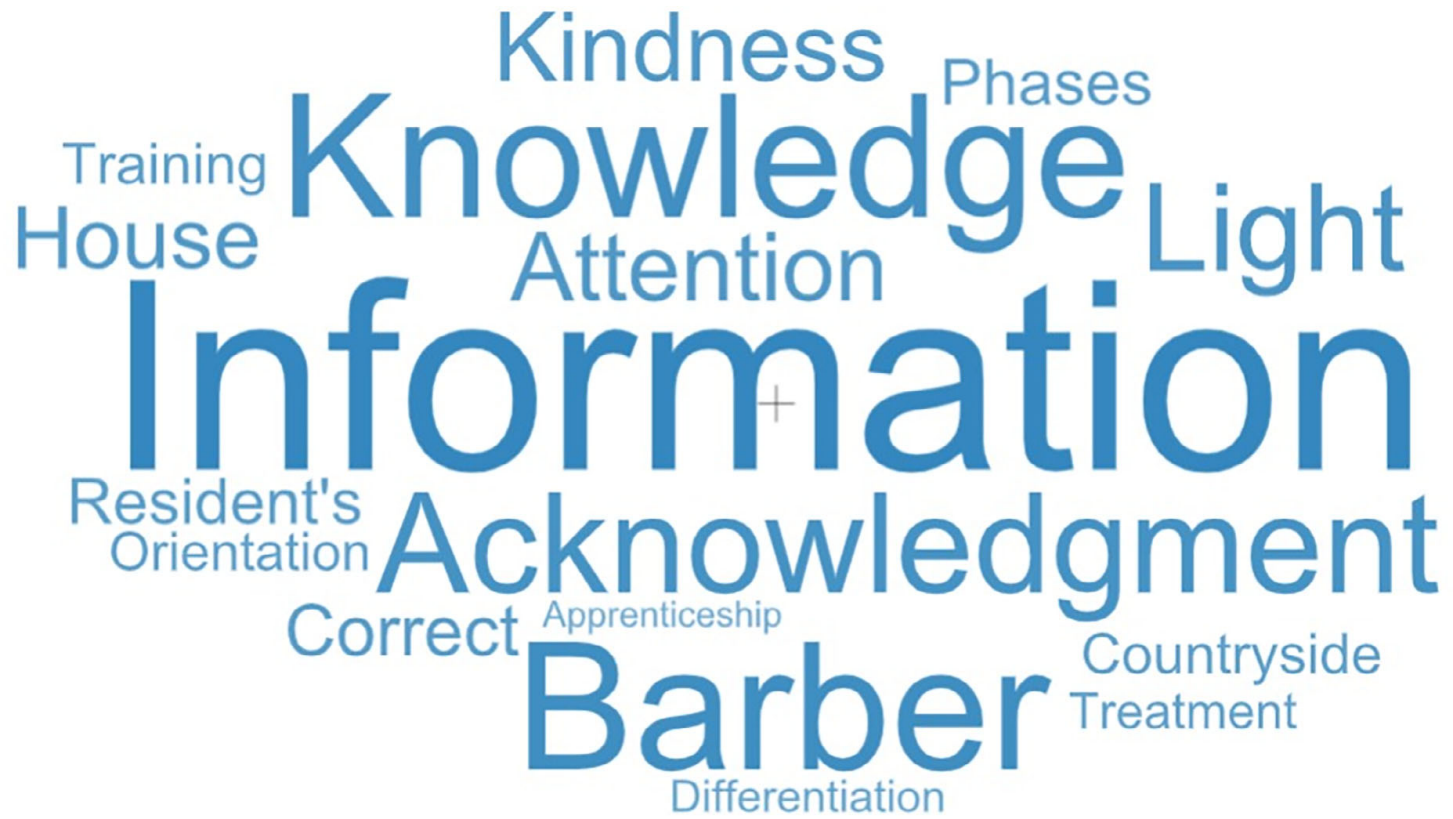
Word cloud built with four statements reported by endemics control agents after participating in wagon 4 educational activities in Chagas Express XXI expedition in Minas Gerais in 2019. *Source*: Cloud prepared in https://wordart.com.

A word cloud with these statements depicted the image in Figure 5, stressing the four main frequent words: information, barber, knowledge, and acknowledgment.

## DISCUSSION

This study is an unprecedented and challenging experience covering the subject in a transdisciplinary way, between the One Health initiative and ArtScience, the sociocultural aspects of endemic areas for CD, and the perception of local knowledge on the subject. This enabled us to build participatory knowledge from the perspective of community engagement, as already discussed in Brazil and other countries [9–11, 29, 34].

The construction of the stands started with the integration of research groups on CD from the Oswaldo Cruz Foundation, including the two institutes hosted in Rio de Janeiro and Belo Horizonte. Over the years, these groups have worked on interpreting the natural phenomena that involve the *T. cruzi* cycle [8, 9, 12], considering that CD is a neglected disease and that afflicts the population mainly in a status of poverty in Latin America. Educational tools become fundamental for the reduction of cases of vector‐borne infection in endemic areas [35].

In this study, the first stand to be presented on the linear path (Stand A) in wagon 4 contained educational materials about the regional CD vectors that were exposed for the population to seek to recognize and to distinguish them from other nonvector insects. Interestingly, some professional agents that work on endemics control reported that it was the first time they observed the triatomines in such a way, as part of the insect's diversity. Wagon 4 was proposed as a site for a deeper immersive experience regarding CD transmission as two preliminary information were offered in the entry “Lassance station” and in the “Discovery and Laboratory” wagon 2 [11]. In the entry stand, a banner presenting the image plates of Carlos Chagas’ original report [36] showed the visitants the *Panstrongylus megistus* published by Chagas in 1909 and further studied by Arthur Neiva. In wagon 2, both hand and bench magnifiers were available for the participant's direct experience of observing the specimens of *Triatoma infestans* in its full cycle stages. We fully understand that both perceptions are important for the knowledge about the vectors: the real scale animals or models, and the magnified images thought lenses or art design. It is not simple for the people to change scales and built their proper unique mental image of the biology elements [37].

In wagon 4, and through the reports of the population on the vectors of the two municipalities, we realized that most of the participants have already had contact with the vectors of the CD, both in the rural area (Espinosa‐MG) and in the urban area (Montes Claros‐MG). However, in another study, it was observed that the knowledge of health professionals about vectors in southern Brazil was not sufficient [38]. The authors found that despite recognizing the vectors 91.7% (*n* = 11), they knew the vectors only indirectly, through explanations, books taught, and magazines [38]. Interestingly for showing large endemic areas and huge populations under infection risk, Argentina and Brazil are leading producers of CD education materials [1, 27, 28]. Nevertheless, the CD is poorly elaborated on Brazilian secondary textbooks, showing confusing and/or insufficient information [39], therefore not reproducing the science related to Carlos Chagas’ legacy. This fact unequivocally indicates that initiatives on health promotion based on education/science popularization are urgently required. Our result showed that the participants have already had contact with the vector and according to the experiences reported, many of these contacts occur since childhood in a home or peridomicile environment.

Considering the evidence that 80 persons answered positively question 7, reporting the observation of vectors in their house environment (Q7, Table 1), we can suggest that the control of the CD vectors does not seem to be effective or does not occur frequently in regions, considering that even the majority of participants in both cities know where to bring the kissing bugs, apparently 80 respondents have seen kissing bugs at their home, from urban and rural areas, where the routine proliferation of kissing bugs occurs, and families are less assisted. Concerning the occurrence of the vectors, this may lead people to discredit on the solutions proposed through the notifications of triatomine encounters to the endemic control services. It then makes even more difficult the control of the vectors. In addition, the need for educational actions on the subject in the region is noticeable, especially in the less‐assisted areas as the recognition of the vector and its phases of development (nymphs—question 2, stand A) are extremely important for the control of CD transmission. The sampled municipalities are within endemic areas, where part of the population is aware of the insects but is unaware of the preventive measures that effectively help in the control of CD. It is essential not only the constant provision of actions of the public health agencies but also more and more community participation, under a situation that overcomes the lack of information [8].

The active and informative methodology of stand B worked using mockups, bringing knowledge about the biology and diversity of the triatomines related to the process of transmission of the agent that causes CD in a playful and dynamic way. This stand shared knowledge to ensure the empowerment of the quality of social life in health within the scope of the One Health concept. One example is the knowledge that changes with the elimination of natural ecotones result in the introduction of many of the species of triatomines that are introduced to the environment at home. The exposure of these dynamics in endemic areas based on both dialog and listening, as advocated [35], leads the population to a better understanding of their reality [40]. In this way, the acquisition of habits that allow impacting the entomological indicators is stimulated, a strategy that is allied in the control actions [7]. This means that a learning provides a comprehensive memory, and a knowledge “links” with what is already known. The children's audience enjoyed retelling what they learned in their own words, a way of sediment knowledge.

Following the constructivist perspective in which teaching‐learning situations are provided, each individual was led to update their mental and affective frameworks and to explain their prejudices, thus managing to build other schemes that are increasingly broad and complex, with greater quantity and quality of interrelationships and more structured [41]. Information related to common activities in endemic areas, such as hunting, caused the greatest impact on the adult population and mainly men when associated with the possible transmission of the parasite by the hunting practices. The women were also concerned as they were responsible for handling and preparing the hunted animals.

In general, the population presented knowledge about the hematophagous diet of the triatomine insect. However, the same was not observed regarding the classic form of transmission of the parasite. Previous study states that the prevention of CD depends primarily on keeping individuals free from contact with the insects that transmit the disease's etiological agent by structuring and maintaining vector surveillance [42]. Once they know the preventive measures and the importance of entomological surveillance, the population will be able to work with the health and endemic professionals in the notifications that will improve the quality of life.

Stand C captured reports that reveal the participants’ perception of the relationship among health, the environment, and wild animals. In this way, our results evidence the lack of knowledge of most of the participants about the participation of wild mammals in the CD cycle. This factor contributes to the vulnerability of this population as many reported being exposed to one or more wild reservoirs species. Stand C was an opportunity to dialog with people from CD endemic regions regarding the animals involved in the maintenance and dispersion of *T. cruzi* in the wild environment as many participants were unaware of the parasite, hosts, and vector cycle. We highlight the needs to develop educational and informational strategies on the *T. cruzi* wild cycle, especially in endemic regions. It was seen that facilitating the presence of possibly contaminated animals close to the home environment represents a health risk [43, 44]. During the activities stand C, most participants reported being exposed to *Didelphidae* marsupials, considered the oldest and most important reservoirs of *T. cruzi* [45], as well as to other important hosts, a risk that is potentialized in areas inhabited by the vector.

For many years, CD was a health problem restricted to rural regions; deforestation is one of the factors that spreads the disease, as it favors the movement of wild host animals and parasite vectors in search of food [45, 46]. This environmental factor reinforces the need for transdisciplinary actions between health education and environmental education, as this one is an essential and permanent component of national education and must be present, in an articulated way, at all levels and modalities of the educational process. Environmental education can collaborate with the One Health approach that advocates an integrated look at health, so it is necessary to think about actions that guarantee the health not only of the human being but also for the ecosystem balance [47].

The stand D exposed the epidemiological importance of the house environment for the CD vectorial transmission given that this environment works as a transition between the wild and home environments, facilitating the entry of triatomines in the home. Although the population living in a rural area (Espinosa‐MG) had greater knowledge of what peridomicile and its components represent as a risk, there is still a low perception that this association allows the entry of wild triatomines in the home and that some conditions of care and organization facilitate the presence of triatomines inside homes. Another fact found in the low perception of the population about the importance of animal shelters and heaps of construction materials. These elements can be housed in an enormous amount of refugee specimens in hiding places generated by cracks and fissures and poor finishing.

The perception changed regarding animals in the peridomicile, and most residents associated the presence of chickens with triatomines, for example. In the urban area (Montes Claros, MG), we noticed that the population had little knowledge about the peridomicile (see Table 1). Those who knew a little more about the subject were either workers in the endemic/vector sector or people who heard reports from family members who lived in rural areas. These different points of view on the epidemiology of CD highlight that it is still seen as a disease that affects only rural areas.

Talking about CD is more than just talking about the disease, it should also explore biological, epidemiological, sociocultural, and political aspects [1]. Following this proposal, we grounded dialogs with participants in stand D, and we were able to immediately (i) exchange and share information, (ii) identify and clarify concepts about this disease through an accessible language based on the technical and scientific knowledge acquired in years of research. The nonformal educational intervention in loco fulfills a primary role in demystifying and clarifying the mistaken knowledge of the populations. In addition, it motivated community engagement in vector surveillance and encouraged residents to notify to public agencies of control of any triatomine finding, thus aiming at improving the quality of life and increasing knowledge about a neglected disease, which still plagues a considerable portion of the population.

With the necessary knowledge, the local population will be in a better position to recognize and act in a participatory way in continuous surveillance actions. Health education is an important initiative that can positively impact the control of endemic diseases. This fact can be corroborated by the systematic review indicating that community participation significantly improves entomological surveillance programs and could be an efficient strategy in the vector control of CD [42].

In the CE XXI expedition, the One Health approach was proposed, and in the fourth wagon, the exhibition was arranged in an environmental trend or *continuum* beginning in the sylvatic environment, with wild animals that may comprise reservoir hosts, to the rural one and ultimately leading to the peridomicile. So, the main steps in the CD transmission chain were displayed and identified by the population in a continued progression. CD became associated with low‐income rural households [49]. Nevertheless, triatomine colonization takes place on wattle and daub houses and in high socioeconomic buildings in urban areas [27, 49–51]. Therefore, in our models, triatomines were placed in diverse types of domiciles to allow a more realistic vision of CD dissemination to the population.

Wagon 4 culminated in the “Virgínia's house” [27] honoring the prominent educator and scientist Virgínia Schall (*in memoriam*). The scenic house crafted in an inflatable igloo follows the sequence of mockups [28], so that the visitors shift from an outsider to an insider point of view, allowing the identification in further detail of the hiding places in the domiciliary and peridomiciliary environment in an immersive and fun experience. Stimulated by provocative questions made by the mediators in a Socratic maieutic method, the participant was empowered to draw their own conclusions. In this activity, the child interaction took place in an active way, playing in a ludic way because playfulness promotes learning [52].

Our health education proposals focus on different endemic infections, mainly neglected diseases [26]. It is worth noting that other infections, including COVID‐19 [53, 54] and intestinal parasites [55, 56], may comprise comorbidities often worsening the clinical conditions of CD‐affected persons. Thus, the One Health view may be instrumental to analyze CD in the intricate scenario of public health particularly of tropical developing nations.

In addition, the control strategies for different vector‐borne infections such as dengue and CD may be implemented simultaneously [57]. In this regard, pathogens are diverse as viruses as SARS‐CoV2, protozoa such as *Entamoeba histolytica* and *Giardia lamblia*; besides, several bacteria may be spread via fecal–oral contaminations. Therefore, sanitary measures can be effective for the prophylaxis of different infectious and parasitic diseases. Similarly, *T. cruzi* may survive for over 24 h in different beverages, including water [58], and therefore may be transmitted by water consumption [28], particularly in semiarid areas, where water is usually stored within households. Thus, the per os contamination may be the infection route for many pathogens, and hygiene procedures may be useful for preventing multiple diseases.

The bona fide One Health view requires the comprehension of households integrating the environment [59]. The presence of wild/domestic animal species at peridomicile may influence CD incidence. The presences of peridomicile dogs, marsupials, and rodents [60, 61, 62], cats [63], chicken coops in the house proximity, and dirt/garbage [64, 65] are considered CD risk factors. For these reasons, intradomicile and peridomicile cleaning and organization can comprise a valuable tool in both leishmaniases [66] and CD [65] control programs. It is worth noting that community participation was shown to be effective in sustained control of triatomine house infestation [62, 68, 69].

As over 10 triatomine species are autochthonous to the southern states of the United States of America, the Center for Disease Control and Prevention informs the population where these vectors can be found in the home environment, although the use of screened windows and doors reduces, considerably the chance of home infestation (https://www.cdc.gov/parasites/chagas/prevent.html). It should be noted that CDC also recommends measures such as organization and cleaning of households and peri‐households to combat vector infestation.

Montes Claros and Espinosa are closely linked with vector‐borne transmission, whereas oral transmission mainly takes place in the Amazon region. In areas where the disease is not endemic, oral transmission is uncommon, particularly when the domestic cycle of triatomines is well‐managed. This situation arises from the exposure of food to infected triatomines and the contaminated secretions of reservoir hosts. In this context, through the use of CE workshops and stands, our focus was on the local scenarios and the development of scientific dissemination strategies that best address the local transmission dynamics of the Chagas parasite within these populations.

The study faced some limitations that are important to acknowledge. First, the linear circuit of the stands might have influenced the participant engagement and data collection, as it restricted the variety of interactions and experiences. The reliance on question‐based methods for evaluation also presents a limitation; alternative approaches, such as using drawings or other creative means, could potentially provide deeper insights into the participants’ understanding and perceptions. Furthermore, although the “One Health” theme is comprehensive, the study could have benefited from incorporating aspects related to domestic animals, which are also relevant for understanding Chagas disease. This could have provided a more holistic view of the disease's impact and transmission dynamics. Lastly, the study's analysis was confined to the preexisting knowledge of the participants, without a follow‐up to track the continuous learning or long‐term impact of the educational interventions on these populations. This limitation restricts the understanding of the effectiveness and longevity of the knowledge imparted through our workshops and stands.

## CONCLUSION

Health promotion strategies, such as CE XXI, are of pivotal importance in CD endemic areas, potentially stimulating population awareness and engagement in therapy as well as in integrated vector management. This study showed that educational materials can be elaborated from the concepts of One Health and ArtScience. The principle of One Health is to integrate several research groups to discuss relevant topics to public health, and in this study, we talk about vectors, hosts, and risk situations presenting artistic–scientific models that contributed to promoting the conversation about prevention and control of CD in rural and urban endemic areas. The population reported contact with several vectors and reservoirs throughout their lives and the presentation model that we carried out allowed participants to relate with several stages that are fundamental to understanding the cycle and the factors that influence the incidence of CD. The interaction between scientists and populations from endemic areas helps to mitigate the risks of CD, as both the scientists need to understand how the spread of this parasite occurs, as well as the population that lives daily with the vectors and reservoirs, family members affected by the disease, become ill due to the lack of knowledge of health and diagnostic services, and of vector control. Therefore, promoting education, prevention, and health promotion should be a priority strategy in dealing with the social, economic, and epidemiological factors of CD.

## AUTHOR CONTRIBUTIONS

*Conceptualization*: Jonathan Gonçalves‐Oliveira, Catarina Macedo Lopes, Teresa C. M. Gonçalves, Suellen Nunes Sarmento, Rute H. A. de Sousa, Antônia de Castro Ribeiro, Otília Sarquis, Ana M. Suarez‐Fontes, Marcos A. Vannier‐Santos, and Tania C. Araujo‐Jorge. *Data curation*: Jonathan Gonçalves‐Oliveira, Catarina Macedo Lopes, Rute H. A. de Sousa, Antônia de Castro Ribeiro, Otília Sarquis, Juliana Almeida‐Silva, Sarah C. dos S. Silva, and Ana M. Suarez‐Fontes. *Funding acquisition*: Tania C. Araujo‐Jorge. *Investigation*: Jonathan Gonçalves‐Oliveira, Catarina Macedo Lopes, Teresa C. M. Gonçalves, Suellen Nunes Sarmento, Rute H. A. de Sousa, Otília Sarquis, Juliana Almeida‐Silva, Sarah C. dos S. Silva, Ana M. Suarez‐Fontes, Marcos A. Vannier‐Santos, and Tania C. Araujo‐Jorge. *Methodology*: Jonathan Gonçalves‐Oliveira, Catarina Macedo Lopes, Teresa C. M. Gonçalves, Otília Sarquis, Ana M. Suarez‐Fontes, Marcos A. Vannier‐Santos, and Tania C. Araujo‐Jorge. *Project administration*: Jonathan Gonçalves‐Oliveira, Roberto Rodrigues Ferreira, Paulo S. D'Andrea, Luciana R. Garzoni, Marcos A. Vannier‐Santos, and Tania C. Araujo‐Jorge. *Resources*: Roberto Rodrigues Ferreira, Paulo S. D'Andrea, Luciana R. Garzoni, Marcos A. Vannier‐Santos, and Tania C. Araujo‐Jorge. *Supervision*: Jonathan Gonçalves‐Oliveira, Roberto Rodrigues Ferreira, Paulo S. D'Andrea, Luciana R. Garzoni, Marcos A. Vannier‐Santos, and Tania C. Araujo‐Jorge. *Validation*: Jonathan Gonçalves‐Oliveira, Catarina Macedo Lopes, Teresa C. M. Gonçalves, Otília Sarquis, Ana M. Suarez‐Fontes, Roberto Rodrigues Ferreira, Marcos A. Vannier‐Santos, and Tania C. Araujo‐Jorge. *Visualization*: Jonathan Gonçalves‐Oliveira, Catarina Macedo Lopes, Teresa C. M. Gonçalves, Suellen Nunes Sarmento, Rute H. A. de Sousa, Antônia de Castro Ribeiro, Otília Sarquis, Juliana Almeida‐Silva, Sarah C. dos S. Silva, Ana M. Suarez‐Fontes, Roberto Rodrigues Ferreira, and Tania C. Araujo‐Jorge. *Writing—original draft preparation*: Jonathan Gonçalves‐Oliveira. *Writing—review and editing*: Jonathan Gonçalves‐Oliveira, Catarina Macedo Lopes, Teresa C. M. Gonçalves, Suellen Nunes Sarmento, João Paulo S. O. Correia, Rute H. A. de Sousa, Antônia de Castro Ribeiro, Otília Sarquis, Juliana Almeida‐Silva, Sarah C. dos S. Silva, Ana M. Suarez‐Fontes, Roberto Rodrigues Ferreira, Paulo S. D'Andrea, Luciana R. Garzoni; Marcos A. Vannier‐Santos, and Tania C. Araujo‐Jorge.

## CONFLICT OF INTEREST STATEMENT

The authors have declared that no conflicts of interest exist.

## ETHICS STATEMENT

The project and all the consent forms and questionnaires were previously analyzed and approved by the Ethics Committee of Research in Humans of the Oswaldo Cruz Institute (CEP‐IOC/Fiocruz, CAAE 15584119.4.0000.5248) according to Brazilian laws and regulations of research with humans, as described previously. This included a formal consent that was obtained of voluntary child participation with two specific instruments, one signed by their parents/guardians and another by the children. In all cases, anonymity was ensured [11].

## Supporting information

Supporting Information

## Data Availability

The authors confirm that the data supporting the findings of this study are available within the article [and/or] its supplementary materials.
