# Supplementary material for: Empowering communities through One Health and ArtScience: An innovative approach to combat Chagas disease in endemic areas of Minas Gerais, Brazil
Source: Public Health Chall. 2025 Feb 10;4(1):e165. doi: 10.1002/puh2.165 (PMC12039351; doi:10.1002/puh2.165)
Supplement: Supplementary file 1 — Supporting Information [file PUH2-4-e165-s001.docx]

**Supplementary data S1 and S2**

**S1.- Description of exhibition stand**

*Stand A – Vector’s morphology and biology*

Stand A used ludic tools to associate the empirical knowledge of local communities about the vectors of *T. cruzi* with formal and scientific knowledge, to explore the morphology and biology of Triatomines. Stand A was composed by (i) two entomological boxes displaying different triatomine life cycle stages, and specimens of other Hemiptera, as well as beetles, and (ii) a banner bringing morphological data about the group (Fig. 2B, Fig. 3A), species of medical interest and guidance on PIT’s (*Posto de Informação de Triatomíneos* – Triatomine Information Posts, Fig. 3A, Fig. 3B). During the activities, two questions were asked to the participants (Fig. 1A:1, 2). The subjects covered: (i) the morphological characters essential for the recognition of triatomines and the differences between their main genera; (ii) the differences between bloodsucking triatomines and other Hemiptera (phytophagous and predators); (iii) why are kissing bugs not beetles ?; (iv) the ways of capturing and handling the insect in a home environment and the relevance of contacting the agencies responsible for controlling endemic diseases (Fig. 3A); (v) and the presentation of the *Triatokey* app (Fig. 3B) (17). To complement the concepts covered, the comic booklets “Barbeiros: Transmissores da Doença de Chagas - Edição 2019” (Kissing bugs: Chagas Disease Vectors) were distributed to participants of different age groups, in addition to the illustrated card packs “Vetores da doença de Chagas no Brasil” (Chagas Disease Vectors in Brazil) and the “Atlas Iconográfico dos Triatomíneos do Brasil: Vetores da Doença de Chagas” (Iconographic Atlas of Brazilian Triatomines: Chagas Disease Vectors) targeted local health workers (18,19).

Stand A commonly referred to the previous experiences of the participants concerning the observation of triatomines in the banner positioned at the entry (Fig. 2 G), with the Carlos Chagas’ original work, as well as in the banner (Fig. 2H) and devices available at Wagon 02, where a plastic Petri dish showed some stages of *Triatoma infestans* to be observed under a hand (Fig. 2I), or a table magnifier (Fig. 2J).

*Stand B – Vector’s ecology*

*Do you know the kissing bugs feed on blood? Do you know how Chagas’ Disease is transmitted?* (Fig. 1B: 3,4) Simple questions that most people in endemic areas should know how to answer correctly. Banners shown in Figures 2C and 2D were used in this stand and a mockup was created representing three different environments, the wild, peridomicile, and the domestic environment inside a house, to present the dynamics of the transmission cycle of the *T. cruzi* parasite in a playful way. In the wild environment, the presence of mammals participating in the wild cycle of the parasite was highlighted, serving as food for the triatomines. Likewise, the importance of possums and rodents that have the habit of invading homes to build their nests. It was emphasized that deforestation and burning, actions related to planting, pasture formation, construction of storerooms, chicken coop, and pigsty, favor the establishment of insects in these new peridomicile environments enabling the formation of triatomine colonies, due to the easy access to different sources of food. In the house environment, in addition to man, dogs and cats are often present, increasing the chances of feeding and colonizing insects. The presence of light in the backyards of the houses is also an attraction factor for insects, contributing to the invasion by flight and enabling the colonization of triatomines. The paths of transmission of the parasite to human beings were highlighted, stressing that the most common in the region is the vectorial transmission that occurs through feces.

*Stand C – Reservoir mammals*

Stand C consisted of an interactive exhibition to show the biodiversity of wild mammals involved in the CD cycle, with the perspective of addressing the potential of certain species in hosting and reserving etiologic agents that cause infections that affect the health of humans. It presented a small collection of taxidermized animals that could be reservoirs for *T. cruzi* in that region and used the support of a specific banner (Fig. 2E) prepared to stimulate a ludic workshop entitled “Biodiversit’Arte” (Biodiversit’Art). In contrast, we also talked about the importance of these animals in maintaining a balanced ecosystem. For this, a problematizing approach was used to stimulate critical sense and awareness and a scientific approach to explore creativity and awareness (9). Two guiding questions were inserted in the dialogue (Fig. 1C: 5,6) and the participant’s observations encouraged them to analyze fundamental points. It is noteworthy that the taxidermized animals played an essential role in this interaction.

The materials used were: taxidermized mammal specimens (Scientific Collection of the Laboratory of Biology and Parasitology of Reservoir Wild Mammal/ LABPMR-IOC/Fiocruz); artistic illustrations produced in watercolor; animal drawings on sheet of paper to color and make masks; a book “Mamíferos do Brasil: Uma Visão Artística” for theoretical complementation (20); illustrative banner composed of theoretical bases on biodiversity and the relationship of wild mammals with CD; the discovery of Carlos Chagas through experiments with wild animals and information on recommendations, what to do when finding wild animals injured or out of their habitat and field notebook the scientific and artistic material cited was selected based on a bibliographic survey of reported wild animals infected by *T. cruzi* and other strains (21).

*Stand D – Peridomicile*

To demonstrate the importance of the home environment for CD transmission, we conceptualized its relevance in the epidemiology of this disease by explaining which conditions favor the arrival and permanence of triatomines. For this, we produced banners and cards with photos of structures that serve as shelters and domestic and synanthropic animals on which triatomines feed. We also produce entomological boxes (in plastic culture plates) containing the development cycle from eggs to adults of *Triatoma brasiliensis*, *Triatoma pseudomaculata*, *Triatoma sordida,* and *Panstrongylus lutzi,* insect species commonly found in the region. Banners in Fig. 2C and Fig. 2D were used in this stand. To assess knowledge about the presence of triatomines, we asked two questions when interacting with visitors (Fig. 1D: 7,8), using banners to explain the attraction characteristics for triatomines showing: (i) animal shelters (pigsty, chicken coop, and perch) that are improperly built or without proper finishing materials that could provide innumerable small spaces that serve as hiding places for triatomines, (ii) the heaps of construction materials placed near or close the walls of the homes, facilitating the entry of triatomines. We carried out dynamics using cards, representing: (a) the various situations of refuge or hiding place for triatomines, using hand portable magnifiers to enlarge and allow the visualization of triatomines present in crevices and breaches, (b) a situation of refuge for triatomines and a correct solution to promote the learning of how we could avoid the presence of triatomines and/or colony formation in the peridomicile.

*Stand E – Dangerous Neighborhood (E1) and Virgínia Schall’s Interactive House (E2)*

Among the activities to popularize science for health promotion, developed by the project “Science on the Road: education and citizenship” (22) two of them were selected to be carried out during the expedition of the Chagas Express XXI, in Wagon 4. The “Dangerous Neighborhood” (E1), was composed of mockup representations of different types of social classes households, with models of furniture, appliance, peridomicile garbage, and other details (23), in which specimens of triatomines were placed, in specific spots that are usually found in houses as, for example, under the platform of sofas and beds, behind pictures and in the small cracks in walls and roofs, as well as representations of the vector in cold porcelain (“biscuit”). A second exhibition was the “Virgínia Schall Interactive House” (E2), which consisted in representing a residence in an inflatable igloo (Fig. 2F) (23), also with specimens of formalin-fixed (dead) triatomines, in specific hidden spots, in addition to others such as under the carpet, in debris (pieces of tiles/bricks, wood and etc.) and garbage accumulated in the home, behind appliances and furniture, such as refrigerator, stove, cupboards and between others. We got some inspiration for this activity in triatomine drawings made by children in our previous studies in Bolivia (24). To assess the previous knowledge of the participants about situations of risk in houses, we proposed two questions for both presentations E1 and E2 (Fig. 1E1, E2: 9,10). Additionally, the activity “Virgínia Schall Interactive House”, addressed the presence of venomous animals inside, as well as vectors of human infections, among other animals (22).

**S2 – Experience reports from the stands**

*Experience reports of Stand A*

During the mediation of educational activities and contact of the participants with the materials, several oral reports emerged about the frequent contact with different kissing bugs species, during childhood, or even throughout the life of different participants and family members. Many speeches exposed the common to cohabitate with insects in the residences in which they live and their precarious conditions. Usually, precarious households provide environments suitable for triatomine infestations. We observed that, for some, this coexistence was ordinary since childhood. Under these conditions, triatomines are easily found under mattresses, on the walls of houses, on the roof, or in the homes of domestic animals. Many participants knew triatomine species but would not be able to recognize their immature phases whether they had already seen them. Some reports stressed that they had already observed the immature phases within intradomicile environments, however, they believed they were other insects. In addition, several participants reported that they could very easily mistake triatomines for other insects, nymphs, or adults, as with some of the specimens exposed in the activity (bedbugs, phytophagous/predator Hemiptera or beetles), emphasizing the importance of knowledge aimed at identifying triatomines. At the end of the activity, the participants received guidance on the collection of specimens in the home and home environment, to be sent to the PIT's spots to be examined, reinforcing the importance of notifying the occurrence and identifying possible triatomine proliferation in the cities, in neighborhoods and mainly in rural areas (Fig. S2.1). The diversity of insect species and characteristics (Fig. S2.1 A-D) were expanded in Wagon 04, after a first triatomine presentation in previous stages of Chagas Express XXI exhibition, as shown in Fig. S2 G-J. The redundancy of this information was deliberate since participants could choose to visit any wagon of the train exhibition and could skip the important observation of triatomine bugs, that was then proposed in Wagons 02 and 04 as an important epidemiological skill to be acquired by the participants.


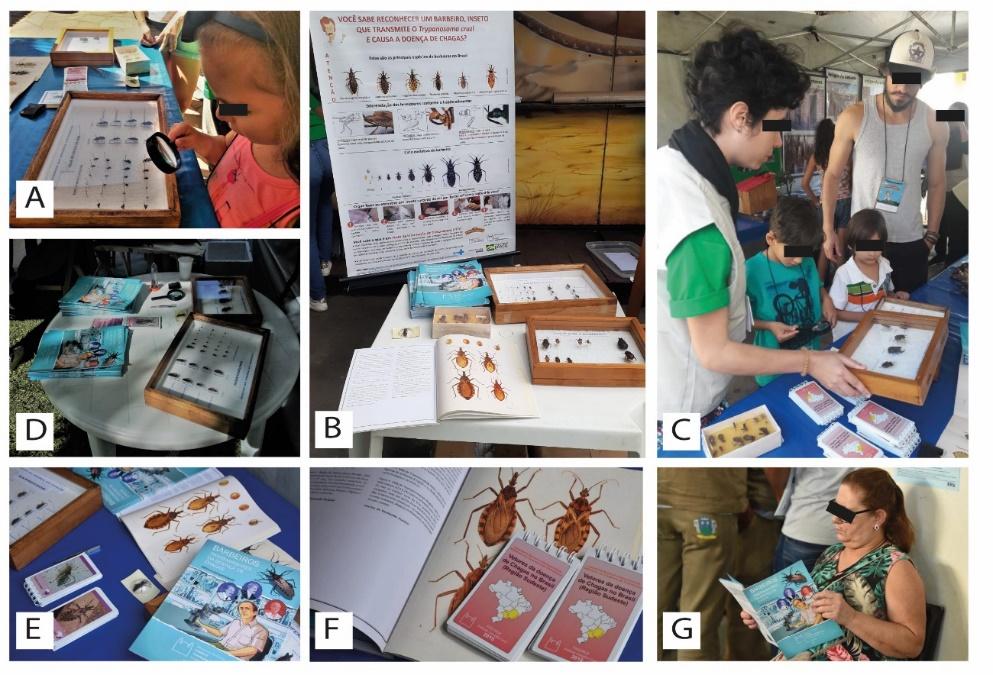


**Figure S2.1:** Activities developed in Stand A of Wagon 4 during the Chagas Express XXI initiative, in the north of Minas Gerais, 2019. Representation (A-F) of the composition of the materials and education resources used during the activities of the Stands, with emphasis on entomological boxes assembled for observation and interaction by the public. (E-F) Layout of the education materials for dissemination that were distributed to the public during the activities, and (G) interaction of the participant with the booklet "Barbeiros: Transmissores da Doença de Chagas - Edição 2019". Photos: Rodrigo Méxas

*Experience reports of Stand B*

The present approach of the social and cultural structure revealed the lack of integrated education and health actions, which weakens the effective prevention of infection in endemic areas, the interruption of active transmission, and makes it impossible to promote awareness and entomological surveillance (Fig. S2.2). In the reports, for most participants, the first contact with these transmitting insects occurred in childhood and continues to be common in the northern region of Minas Gerais. In responses to the questions, we observed that the knowledge of the population is built on a misconception, which relates to the transmission of the *T. cruzi* parasite, with the insect blood meal. Once they learned the form of contagion via contact with the insect feces, most people got surprised. Surprisingly for our team, many health professionals expressed doubts regarding the different forms of contagion.

We also presented the information on anthropic actions modifying the environment, an imbalance factor that promotes the dispersion process of triatomines and vertebrate animals. Likewise, information about the occurrence of the parasite natural cycle transmission among various wild animals drew attention and surprised the participants, since there were frequent reports of the practice of fauna hunting in addition to handling and eating meat raw.

It was worrisome the lack of notification by the residents of the insect findings in the house environment and the invasion of the insect in the municipality’s Endemic Department. We observed reports regarding the lack of feedback actions by the health workers from this sector, as well as no incentive for this practice.

**
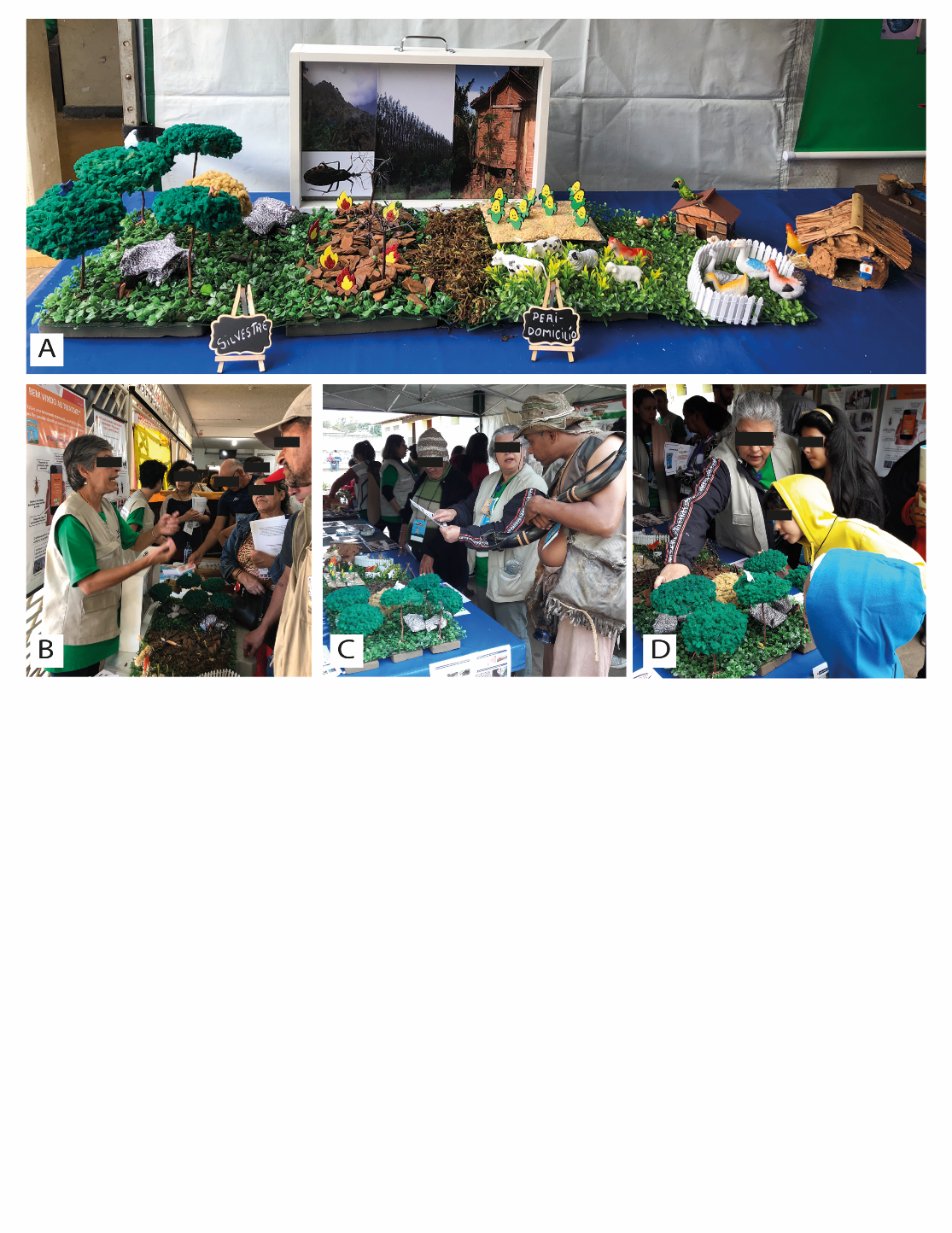
**

**Figure S2.2**: Activities developed in stand B of Wagon 4 during the Chagas Express XXI initiative, in the north of Minas Gerais, 2019. (A) Exhibition model of the environment and the changes that occurred and the influence on the mammal fauna and insects; (B) Exposition of the theme for community health agents; (C) Exhibition of the theme for the Public; (D) Exhibition for children. Photos: Catarina Macedo & Teresa Cristina Gonçalves.

*Experience reports of Stand C*

The exposure of small wild mammals in Brazil aroused wide curiosity (Fig. S2.3). The diversity of this fauna was not widely known among the participants, from which the recognition of each species and its features was made. Most participants reported the presence of one or more species in their domiciles and/or surroundings, in chicken coops, roofs, trees in the yard. The most cited mammals were precisely those with the greatest potential to act as reservoirs: possum, marmosets, rats, and armadillos. Such exposure to *T. cruzi* reservoirs is alarming since the region is endemic to the vector involved in the CD cycle. Reports, including some from health professionals, revealed that host animals are not clearly associated with the disease, thus ruling out the perception of risk. Even domestic animals like dogs and cats were not known as potential *T. cruzi* reservoirs or blood-sucking sources for triatomines. The animal most described as seen in the home environment was the possum, in view of which the stand clarified about the possibility of infection even though remote through its urine, information that was received with great surprise. The participation of hunters who questioned the possibility of infection by eating meat was timely to warn about the risks and precautions when handling wild animals and the importance of these for a balanced ecosystem. It was also clarified that only mammals have the potential to harbor the *T. cruzi*, as many participants believed that chickens could transmit the disease.

The artwork of wild animal masks available to color and to cut stimulated creativity and awareness about the importance of biodiversity (Fig. S2.3 B-D). The stand offered and received information about local customs, attitudes, and empirical knowledge of residents of rural and urban areas regarding the wild animals of the biomes of that region. We consider an advance in the popularization of the Brazilian fauna of small wild mammals contributing to the valorization of our biodiversity and in the popularization of the relevance of developing scientific research involving this group.


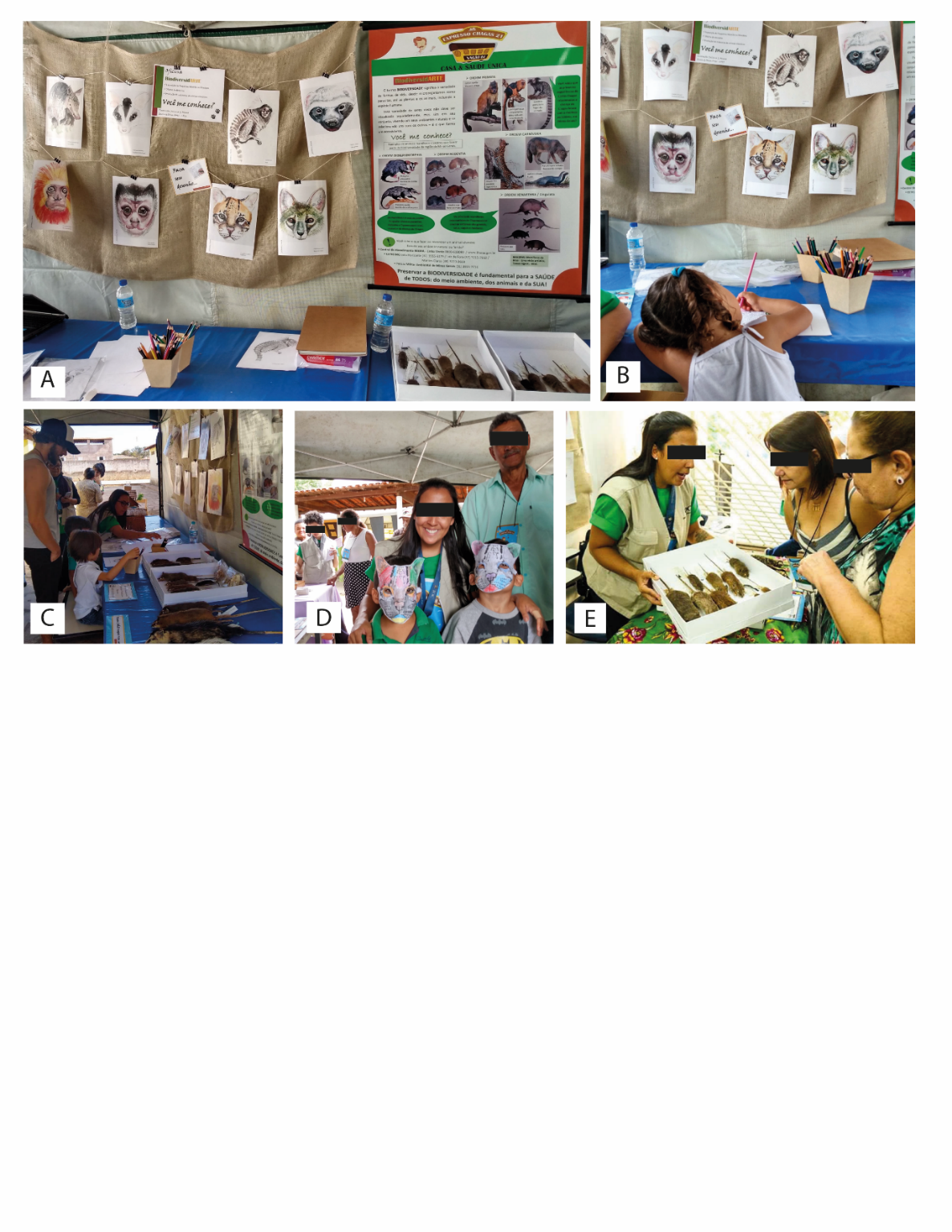


**Figure S2.3**: Activities developed in stand C of Wagon 4 during the Chagas Express XXI initiative, in the north of Minas Gerais, 2019. Education materials used in Stand C (A). Interaction with participants through critic dialogues through education, scientific and artistic materials (B-E). Photo: Rodrigo Méxas.

*Experience report of Stand D*

When asked about the home location of triatomines, all participants knew how to identify the spots, often relating it to the yard, and when requested, they pointed out the species *T. brasiliensis* and *T. sordida* as the most often locally seen. Concerning the shelters, many participants were amazed at the refuges and the number of triatomines found (Fig. S2.4). They also did not realize that they could transport triatomines to their homes through wood or stuck to their clothes. We emphasized attention to the choice of material for construction and care with the finishing of shelter, in addition to cleaning the land. This fact became more interesting within the search for triatomines at the figures that showed different refuges and children were the most excited about this activity. About animals, many participants were unaware that wild animals in the peridomicile could carry triatomines there and even indoors. They were also unaware that peridomestic and domestic animals attract triatomines that feed upon them. Some participants associated the presence of the triatomine only with the existence of chickens, which caused them concern and fear of being contaminated when feeding on the bird. However, when informed of the refractoriness, explained in a simple and easy-to-understand manner, they showed relief and satisfaction in acquiring such knowledge. Participants were advised to avoid raising animals at home, a common habit among rural men. Throughout the activities, we noted the disinclination of the participants regarding the delivery of triatomines found in the peridomicile; the lack of information on procedures to collect and store triatomines, and how to notify the competent authorities were also reported. The participation of agents of endemics control provided a lot of knowledge exchange, as they reported their real experiences and how the work proceeds as we talked about the biology and ecology of triatomines.


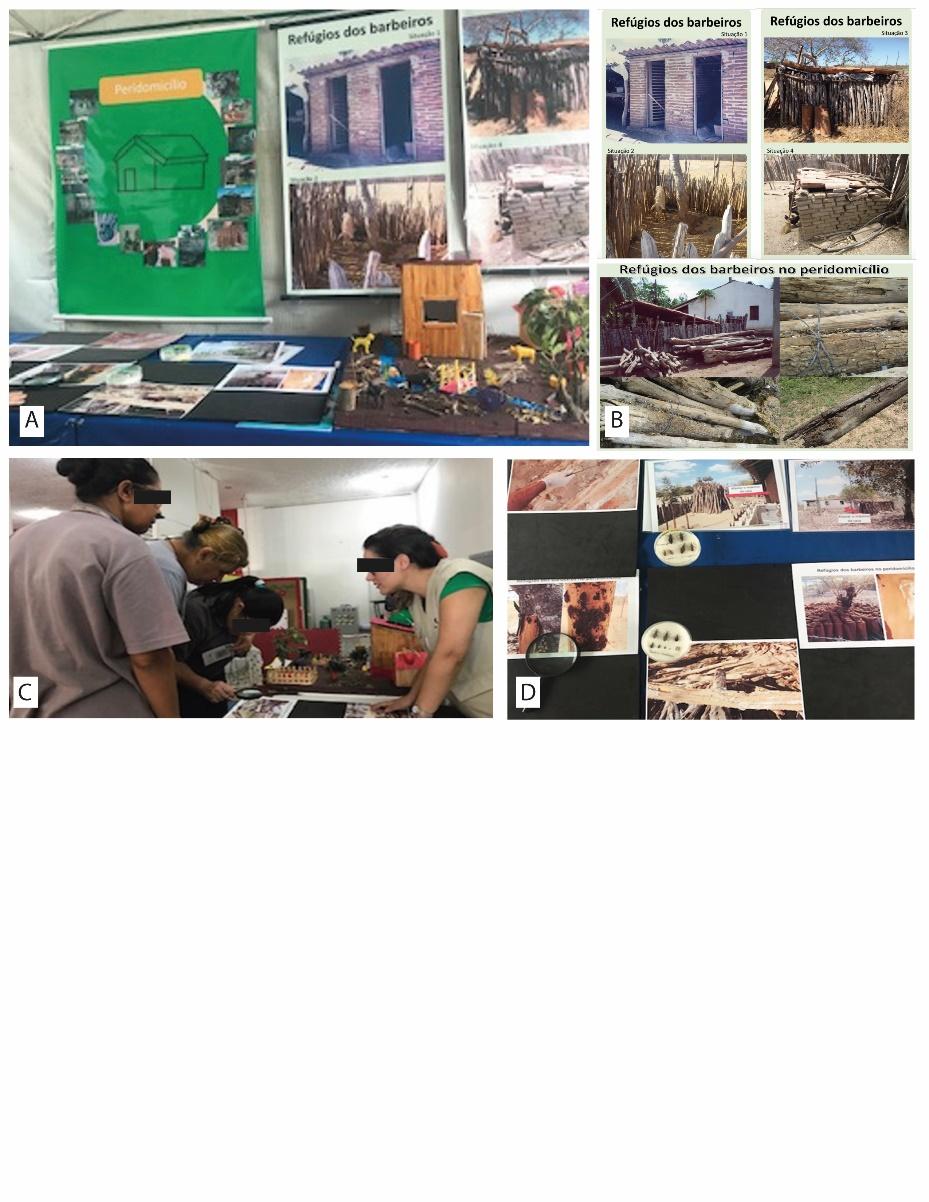


**Figure S2.4:** Activities developed in Stand D of Wagon 4 during the Chagas Express XXI initiative, in northern Minas Gerais, 2019. Representation (A-D) of the composition of materials and educational resources used in the activities. Photos: Otília Sarquis

*Experience report of Stands E1 and E2*

Concluding the education circuit proposed by Wagon 4, the last stand was composed of two activities, developed by the program “Science on the Road: education and citizenship”, which aimed to focus on the potential sites of infestation by triatomines in the home environment. In addition, other common infections and breeding sites for their vectors were addressed, in addition to venomous animals, constituting an environment with different ways of promoting health.

In the first activity of the Stand (E1) “Dangerous Neighborhood” (Fig. S2.5 A-F), some visitors reported previous knowledge about the vector and its distribution, quite possibly due to everyday experiences, as well as work experience since community health agents enthusiastically participated in the activity. Many participants reported that during childhood, they eventually feared falling asleep at night when they noticed large amounts of triatomines on the walls and roof of the residence. In the interactions and dialogues, it was possible to notice the happy expression of the visitors when observing the model represented houses and the manifested feeling of belonging when recognizing types of houses. This first stage of this stand reached great representativeness, identification and belonging with the experienced reality. Excited expressions were observed in view of the sharing of new information presented during the mediation.

The last stage of the stand (E2) consisted of a visit to the scenic device “Virgínia Schall’s Interactive House” (Fig. S2.5 G-K). However, not all participants who went through the first exhibition (E1) went on to this activity, making it impossible to measure the learning accomplished by each activity. In the interactive house, triatomines were positioned under beds and sofas, inside the cracks in walls, roofs, and other spots. During the expedition, it was possible to discuss real reports and some participants who claimed to have found triatomines under carpets, a fact not found in the scientific literature. This exchange of experiences made it possible to enrich the activity, bringing the event closer to the population of the endemic area, in the context of CD. In previous activities of the “Science on the Road” program, we were informed by health agents about the transmission of *T. cruzi* by ingesting stored and contaminated water at home, especially in semi-arid regions (25). Once again, contact with residents provided us with information that may be relevant for future activities, as well as for the students' experience.

During the visit the interactive house constitutes a wider and more comprehensive universe, inducing the resizing and sense of belonging, which reveals to the visitor’s aspects unnoticed in the models. During the visits, the participants were invited to simulate a sort of “treasure hunt”, searching for triatomines hidden in strategic locations (Fig. S2.5 I-J), depending on the use of the domiciliary environment. Participants directly probed common places, such as bed, sofa, and wall and, with the supervision of mediators, they discovered, through tips and questions, the other possible places of shelter, as in debris, rubbish, and accumulated materials, behind furniture and appliances.

In both activities, the presence and interaction of visitors from different age groups were noted. In both cities visited, the participation of children was daily and took place full-time. Some called their friends and neighbors, and each day, a new visitor appeared to compose the group, in such a way that the first to visit the stand was already able to mediate the education trail for their new attendees as information multipliers enhancing activity effectivity (22,23). Therefore, stands E1 and E2 were considered attractive, which allows for a more comprehensive infection prophylaxis work, reaching a wide variety of individuals. This raises a very important point, which is the ease of reproducing and dissemination the information acquired through mediations, in order to gradually form “health promotion movements” engaging the communities for potentially contributing to the prevention of CD cases in the region.

The mediations included information about the care that should be taken inside houses, as well as the accumulation of materials and garbage in the external areas, which are potential attractions for cockroaches, rodents, venomous animals, and vectors of various infections, in addition to polluters of the environment. Thus, procedures were recommended, such as avoiding the incorrect disposal of waste in watercourses and soil, following the concept of One Health, the central subject of this wagon.


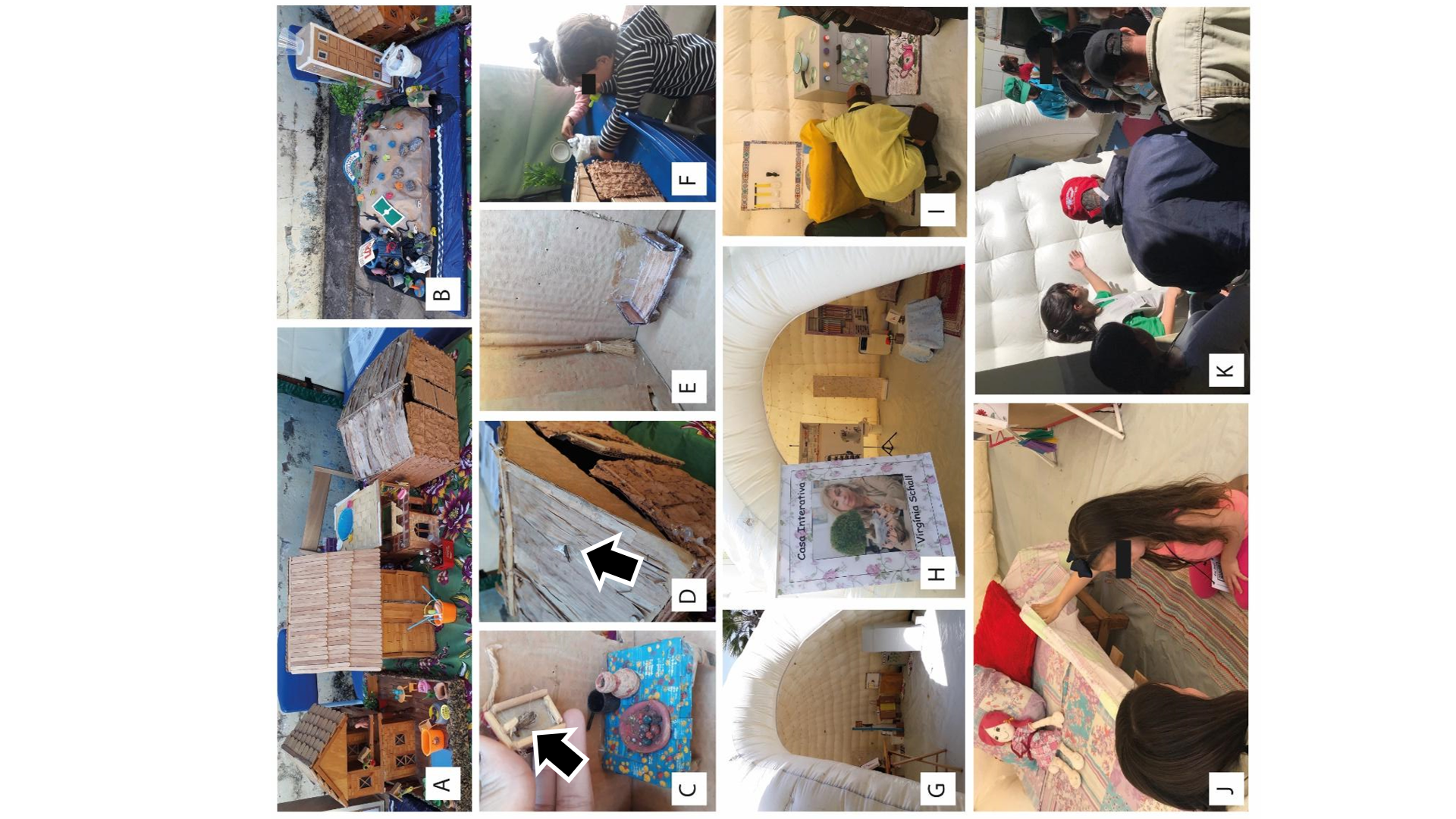


**Figure S2.5**: Activities developed by the team of the “Science on the Road: education and citizenship” program, in Wagon 4 of Chagas Express XXI, in Minas Gerais, 2019. Represented “Dangerous Neighborhood” (A-F), displaying triatomine infestation in houses of diverse socioeconomical levels (A) and the anthropized landscape (B) showing, incorrectly disposed trash, sewer and other details. Fixed triatomines were placed within the mockups, behind a picture and on the roof photos inside the models (Figures C, D - arrows), under coach sofa (E) and elsewhere. Children spontaneously simulated cleaning the yards, collecting the trash (F), indicating possible protagonist stimulation. The domicile milieux was also scenic represented in inflatable igloo, a device named “Virgínia Schall’s Interactive House” (G, H). Many triatomine hiding places, were searched by the visitors, including under/behind appliances (I) and under the bed frame (J) etc. There were participants from different age groups searching for the triatomines shelters within the house (I, J) in addition to the peridomicile (K). Photos: Science on the Road program collection.

**S3.- Knowledge of children and teenagers participating in wagon 4 during educational activities**

|  |  | **Espinosa (total of children 6-17 years old participants = 79; 19% of all participants - 411)** | | | | | | | | | | | | | | |
| --- | --- | --- | --- | --- | --- | --- | --- | --- | --- | --- | --- | --- | --- | --- | --- | --- |
|  |  | Espinosa Pre-school (aged <6 years old) | | | | | Espinosa Elementary (aged 6-14 years old) | | | | | Espinosa High School (aged 15-17 years old) | | | | |
| **Category** | **Table 2: Children from Espinosa** | **Age*** | **Respondents n (%)**  **100% = 79** | **Answers Yes** | **Answers No** | **No Answer** | **Age*** | **Respondents n (%)**  **100% = 79** | **Answers Yes** | **Answers No** | **No Answer** | **Age*** | **Respondents n (%)**  **100% = 79** | **Answers Yes** | **Answers No** | **No Answer** |
| Transmission | A: Q1-Do you know the kissing bugs? |  | 0 | 0 | 0 | 8 | 11.3 | 6 (7) | 2 | 4 | 53 | 16 | 2 (2,5) | 2 | 0 | 10 |
| Transmission | A: Q2-Do you now the kissing bugs´nymphs? |  | 0 | 0 | 0 | 8 |  | 6 (7) | 0 | 6 | 53 |  | 2 (2,5) | 1 | 1 | 10 |
| Control | B: Q3-Do you know the kissing bugs feed on blood? | 4.2 | 6 (7) | 3 | 3 | 2 | 9.8 | 36 (45) | 22 | 14 | 23 | 16 | 2 (2,5) | 2 | 0 | 10 |
| Transmission | B: Q4-Do you know how Chagas disease is transmitted? |  | 6 (7) | 5 | 1 | 2 |  | 36 (45) | 10 | 26 | 23 |  | 2 (2,5) | 0 | 2 | 10 |
| Control | C: Q5-Did you find any mammals like that, close to your home? |  | 0 | 0 | 0 | 8 | 11.3 | 24 (30) | 15 | 9 | 35 | 16 | 7 (8) | 5 | 2 | 5 |
| Transmission | C: Q6-Do you know what the relation of these mammals with Chagas disease? |  | 0 | 0 | 0 | 8 |  | 24 (30) | 2 | 22 | 35 |  | 7 (8) | 6 | 1 | 5 |
| Transmission | D: Q7-Have you ever seen the kissing bugs in a shelter close to your home? |  | 0 | 0 | 0 | 8 | 11.4 | 10 (13) | 0 | 10 | 49 |  | 0 | 0 | 0 | 12 |
| Control | D: Q8-Do you know where to take the kissing bugs that were found? |  | 0 | 0 | 0 | 8 |  | 10 (13) | 3 | 7 | 49 |  | 0 | 0 | 0 | 12 |
| Transmission | E1: Q9-Do you know where the kissing bugs can hide in the house? | 4 | 1 (1) | 0 | 1 | 7 | 8 | 11 (14) | 9 | 2 | 48 | 15 | 2 (2,5) | 2 | 0 | 10 |
| Control | E1: Q10-Does the kissing bugs prefer to take shelter in light of dark places? |  | 1 (1) | 0 | 1 | 7 |  | 11 (14) | 8 | 3 | 48 |  | 2 (2,5) | 1 | 1 | 10 |
| Transmission | E2: Q9-Do you know where the kissing bugs can hide in the house? | 3.6 | 3 (3) | 1 | 2 | 5 | 9.5 | 23 (29) | 10 | 13 | 36 | 15.5 | 4 (5) | 2 | 2 | 8 |
| Control | E2: Q10-Does the kissing bugs prefer to take shelter in light of dark places? |  | 3 (3) | 2 | 1 | 5 |  | 23 (29) | 15 | 8 | 36 |  | 4 (5) | 3 | 1 | 8 |
|  | Total number of answers (100%) |  | 20 (100) | 11 (55) | 9 (45) |  |  | 220(100) | 96(43) | 124(57) |  |  | 34 (100) | 24 (70) | 10 (30) |  |
|  | Transmission related knowledge (7 questions) |  | 10 (100) | 6 (60) | 4 (40) |  |  | 116 (100) | 33 (28,5) | 83 (71,5) |  |  | 19 (100) | 13 (69) | 6 (31) |  |
|  | Control related knowledge (5 questions) |  | 10 (100) | 5 (50) | 5 (50) |  |  | 104 (100) | 63 (60,5) | 41 (39,5) |  |  | 15 (100) | 11 (64) | 4 (36) |  |

Table 1 – Answers of Children from Espinosa, Minas Gerais

* age (mean)

Table 2 - Answers of Children from Montes Claros, Minas Gerais

|  |  | **Montes Claros (total of children 6-17 years old participants = 9; 3% of all participants - 238 )** | | | | | | | | | | | | | | |
| --- | --- | --- | --- | --- | --- | --- | --- | --- | --- | --- | --- | --- | --- | --- | --- | --- |
|  |  | Montes Claros Pre-school (aged <6 years old) | | | | | Montes Claros Elementary (aged 6-14 years old) | | | | | Montes Claros High School (aged 15-17 years old) | | | | |
| **Category** | **Table 2: Children from Montes Claros** | **Age*** | **Respondents n (%) 100% = 9** | **Answers Yes** | **Answers No** | **No Answer** | **Age*** | **Respondents n (%) 100% = 9** | **Answers Yes** | **Answers No** | **No Answer** | **Age*** | **Respondents n (%) 100% = 9** | **Answers Yes** | **Answers No** | **No Answer** |
| Transmission | A: Q1-Do you know the kissing bugs? |  | 0 | 0 | 0 | 0 | 12 | 1 (11) | 0 | 1 |  |  | 0 | 0 | 0 |  |
| Transmission | A: Q2-Do you now the kissing bugs´nymphs? |  | 0 | 0 | 0 | 0 |  | 1 (11) | 0 | 1 |  |  | 0 | 0 | 0 |  |
| Control | B: Q3-Do you know the kissing bugs feed on blood? |  | 0 | 0 | 0 | 0 | 11.5 | 2 (22) | 2 | 0 |  |  | 0 | 0 | 0 |  |
| Transmission | B: Q4-Do you know how Chagas disease is transmitted? |  | 0 | 0 | 0 | 0 |  | 2 (22) | 2 | 0 |  |  | 0 | 0 | 0 |  |
| Control | C: Q5-Did you find any mammals like that, close to your home? | 6 | 1 (11) | 0 | 1 | 0 | 10 | 7 (77) | 5 | 2 |  | 10 | 1 (11) | 1 | 0 |  |
| Transmission | C: Q6-Do you know what the relation of these mammals with Chagas disease? |  | 1 (11) | 0 | 1 | 0 |  | 7 (77) | 0 | 7 |  |  | 1 (11) | 1 | 0 |  |
| Transmission | D: Q7-Have you ever seen the kissing bugs in a shelter close to your home? |  | 0 | 0 | 0 | 0 | 12 | 1 (11) | 0 | 1 |  |  | 0 | 0 | 0 |  |
| Control | D: Q8-Do you know where to take the kissing bugs that were found? |  | 0 | 0 | 0 | 0 |  | 1 (11) | 1 | 0 |  |  | 0 | 0 | 0 |  |
| Transmission | E1: Q9-Do you know where the kissing bugs can hide in the house? |  | 0 | 0 | 0 | 0 | 10 | 3 (33) | 2 | 1 |  |  | 0 | 0 | 0 |  |
| Control | E2: Q10-Does the kissing bugs prefer to take shelter in light of dark places? |  | 0 | 0 | 0 | 0 |  | 3 (33) | 1 | 2 |  |  | 0 | 0 | 0 |  |
| Transmission | E1: Q9-Do you know where the kissing bugs can hide in the house? |  | 0 | 0 | 0 | 0 | 10 | 1 (11) | 1 | 0 |  |  | 0 | 0 | 0 |  |
| Control | E2: Q10-Does the kissing bugs prefer to take shelter in light of dark places? |  | 0 | 0 | 0 | 0 |  | 1 (11) | 1 | 0 |  |  | 0 | 0 | 0 |  |
|  | Total number of answers |  | 2 (100) | 0 | 2 | 0 |  | 30 (100) | 15 (50) | 15 (50) | 0 |  | 2 (100) | 2 (100) | 0 |  |
|  | Transmission related knowledge (7 questions) |  | 1 (100) | 0 | 1(100) | 0 |  | 16 (100) | 5 (31) | 11 (69) |  |  | 1 (100) | 1 (100) |  |  |
|  | Control related knowledge (5 questions) |  | 1 (100) | 0 | 1 (100) | 0 |  | 14 (100) | 10 (71) | 4 (29) |  |  | 1 (100) | 1 (100) |  |  |
|  |  |  |  |  |  |  |  |  |  |  |  |  |  |  |  |  |
